# Supplementary material for: Assessing the extent of drug interactions among patients with multimorbidity in primary and secondary care in the West Midlands (UK): a study protocol for the Mixed Methods Multimorbidity Study (MiMMS)
Source: BMJ Open. 2017 Sep 18;7(9):e016713. doi: 10.1136/bmjopen-2017-016713 (PMC5623557; doi:10.1136/bmjopen-2017-016713)
Supplement: Supplementary file 1 [file bmjopen-2017-016713supp001.pdf]

Supplementary file 1 Read codes

Condition	Included (including exception reporting)	Excluded
Hypertension	G2...<br>G20..%<br>G24.. - G2z.. (Excluding G24z1, G2400, G2410, G27..)<br>Gyu2.<br>Gyu20	21261<br>212K.
COPD	H3…<br>H31..% (excluding H3101, H31y0, H3122)<br>H32..%<br>H36.. - H3z.. (excluding H3y0., H3y1.)<br>H4640<br>H4641<br>H5832<br>Hyu30<br>Hyu31	2126F
Depression	E0013,<br>E0021<br>E112.%,<br>E113.%<br>E118.,<br>E11y2<br>E11z2,<br>E130.<br>E135.<br>E2003<br>E291.,<br>E2B..<br>E2B1.,<br>Eu204<br>Eu251<br>Eu32.% (excluding Eu32A, Eu32B, Eu329) Eu33.%<br>Eu341<br>Eu412	212S.
Diabetes	C10..,<br>C109J,<br>C109K,<br>C10C.,<br>C10D.,<br>C10E.%,<br>C10F.% (Excluding C10F8),	21263<br>212H.

	C10G.%,<br>C10H.%,<br>C10M.%,<br>C10N.%,<br>PKyP.,<br>C10P.%,<br>C10Q.	
Hypertension	G2...<br>G20..%<br>G24.. - G2z.. (Excluding G24z1, G2400, G2410, G27..)<br>Gyu2.<br>Gyu20	21261<br>212K.
Osteoarthritis	N05..00 Osteoarthritis and allied disorders	
CKD	1Z12.<br>1Z13.<br>1Z14.<br>1Z15.<br>1Z16.<br>1Z1B. - 1Z1L.<br>1Z1T.<br>1Z1V.<br>1Z1W.<br>1Z1X.<br>1Z1Y.<br>1Z1Z.<br>1Z1a.<br>1Z1b.<br>1Z1c.<br>1Z1d.<br>1Z1e.<br>1Z1f.<br>K053.<br>K054.<br>K055.<br>1Z10.<br>1Z11.<br>1Z17. - 1Z1A.<br>1Z1M.<br>1Z1Q.<br>K051.<br>K052.<br>1Z1N.<br>1Z1P.<br>1Z1R.<br>1Z1S.	2126E
