# Supplementary material for: Assessing the extent of drug interactions among patients with multimorbidity in primary and secondary care in the West Midlands (UK): a study protocol for the Mixed Methods Multimorbidity Study (MiMMS)
Source: BMJ Open. 2017 Sep 18;7(9):e016713. doi: 10.1136/bmjopen-2017-016713 (PMC5623557; doi:10.1136/bmjopen-2017-016713)
Supplement: Author's manuscript [file bmjopen-2017-016713.draft_revisions.pdf]

BMJ Open is committed to open peer review. As part of this commitment we make the peer review history of every article we publish publicly available.

When an article is published we post the peer reviewers' comments and the authors' responses online. We also post the versions of the paper that were used during peer review. These are the versions that the peer review comments apply to.

The versions of the paper that follow are the versions that were submitted during the peer review process. They are not the versions of record or the final published versions. They should not be cited or distributed as the published version of this manuscript.

BMJ Open is an open access journal and the full, final, typeset and author-corrected version of record of the manuscript is available on our site with no access controls, subscription charges or pay-per-view fees (http://bmjopen.bmj.com).

If you have any questions on BMJ Open's open peer review process please email info.bmjopen@bmj.com

BMJ Open

Assessing multimorbidity in primary and secondary care: A study protocol for the Mixed Methods Multimorbidity Study (MiMMS)

Journal:	*BMJ Open*
Manuscript ID	bmjopen-2017-016713
Article Type:	Protocol
Date Submitted by the Author:	03-Mar-2017
Complete List of Authors:	Backman, Ruth; University of Birmingham, Institute of Applied Health Research<br>Weber, Philip; University of Birmingham, School of Computer Science<br>Turner, Alice; University of Birmingham, Institute of Inflammation & Aging; Heart of England NHS Foundation Trust<br>Lee, Mark; University of Birmingham, School of Computer Science<br>Litchfield, Ian; University of Birmingham, Institute of Applied Health Research
**Primary Subject Heading**:	Medical management
Secondary Subject Heading:	Evidence based practice, Qualitative research, Pharmacology and therapeutics
Keywords:	Protocol, Multimorbidity, Polypharmacy, NHS, PRIMARY CARE, SECONDARY CARE

SCHOLARONE™
Manuscripts

For peer review only

Assessing multimorbidity in primary and secondary care: A study protocol for the Mixed Methods Multimorbidity Study (MiMMS)

Ruth Backman[1], Philip Weber[2], Alice M Turner[3,4], Mark Lee[2], Ian Litchfield[1‡].

[1] Institute of Applied Health Research, University of Birmingham, Edgbaston, Birmingham B15 2TT

[2] School of Computer Science, University of Birmingham, Edgbaston, Birmingham B15 2TT

[3] Heartlands Hospital, Heart of England NHS Foundation Trust, Bordesley Green East, Birmingham B9 5SS

[4] Institute of Inflammation & Aging, University of Birmingham, Edgbaston, Birmingham B15 2WB

[‡] Corresponding author

Address: Institute of Applied Health Research, College of Medical and Dental Sciences
University of Birmingham, Edgbaston, Birmingham B15 2TT
United Kingdom

E-mail: i.litchfield@bham.ac.uk

Tel: +44 (0)121 414 6006

Word count: 3094

Word count (Abstract): 275

For peer review only

ABSTRACT

Introduction: The numbers of patients with three or more chronic conditions (multimorbidity) are increasing, and will rise to 2.9 million by 2018 in the United Kingdom alone. Currently in the UK, conditions are mainly managed using over 250 sets of single condition guidance, which has the potential to generate conflicting recommendations for lifestyle and concurrent medication for individual patients with more than one condition. To address some of these issues, we are developing a new computer-based tool to help manage these patients more effectively. For this tool to be applicable and relevant to current practice we must first better understand how existing patients with multimorbidity are being managed, particularly relating to concerns over prescribing and potential polypharmacy.

Methods and analysis: Up to four secondary care centres, two community pharmacies and between four to eight primary care centres in the West Midlands will be recruited. Interviewees will be purposively sampled from these sites, up to a maximum of 30. In this mixed methods study (MiMMS), we will perform a dual framework analysis on the qualitative data; the first analysis will use the Theoretical Domains Framework to assess barriers and enablers for healthcare professionals around the management of multimorbid patients; the second analysis will use Normalisation Process Theory to understand how interventions are currently being successfully implemented in both settings. We will also extract quantitative anonymised patient data from primary care to determine the extent of polypharmacy currently present for patients with multimorbidity in the West Midlands.

For peer review only

Ethics and Dissemination: Favourable ethical approval has been granted by The University of Birmingham Research Ethics Committee. Our work will be disseminated through peer reviewed literature, trade journals, and conferences. We will also use a dedicated web page hosted by the University to serve as a central point of contact and a repository of our findings.

Protocol registration: NIHR Clinical Research Network Portfolio Registration CPMS ID 30613.

Strengths and limitations of this study:

- Using a mixed methods approach will be able to elicit current barriers found within both primary and secondary care when managing patients with multimorbidity and polypharmacy and so provide context to the prescribing data.
- This is the first quantitative exploration of polypharmacy in multimorbid patients in UK primary care. It is using novel software to interrogate routinely collected prescribing data.
- This is a pilot study only using a small number of urban sites within the West Midlands so the barriers found may not be fully generalisable across the UK. However, the study practices will be purposively selected to represent a variety in size and patient demographic.
- The novel software developed for this study is currently only compatible with EMIS Health clinical system. Other clinical management systems may have different functionalities that allow the more effective management of multi-morbid patients.
-

For peer review only

Keywords: Protocol; Multimorbidity; Polypharmacy; NHS; Primary care; Secondary care.

INTRODUCTION

In the United Kingdom (UK) there is a focus on the formation and implementation of guidelines to help standardise and improve the quality of care.[1-3] Providers may choose to translate this guidance into local policy to help implementation, for example, a multidisciplinary care plan which details essential steps in the management of patients with a specific clinical problem.[4] These pathways frequently use graphical descriptions of evidence and options and are typically represented in a single, or series of, flow charts[5,6] and have been shown to have the possibility to improve care if they are implemented correctly.[7] These systems will contain prompts or reminders which in isolation can help to improve practice[8-10] but in combination can lead to alert fatigue.[11]

Of the 250 clinical guidelines published by the National Institute for Clinical Excellence (NICE),[12] only four account for more than one condition. This presents issues for clinicians attempting to use this guidance to manage multimorbidity. Defined as two or more chronic conditions in the same individual,[13] patients with multimorbidity are becoming more prevalent and it is estimated that by 2018 the number of people in the UK with three or more long term conditions will have grown to 2.9 million.[14] Using multiple single conditions guidelines means patients can be left with a significant treatment burden[15,16] and conflicting advice around lifestyle factors.[17] There are also implications for clinicians who struggle to balance the requirements of several guidelines for multimorbid patients, hindered by the lack of outcome data.[18]

For peer review only

One significant consequence of following multiple guidelines is the concurrent prescribing of multiple medications. This phenomenon has been termed “polypharmacy”[19] and has been identified as a complicating issue for patients with multimorbidity.[20] Polypharmacy is an inevitable part of following many sets of guidance in patients with multimorbidity[21] and healthcare professionals recognise that fulfilling multiple guidelines might compromise patient centred care.[22] There is evidence this simultaneous medication administration can lead to an increased risk of patient hospitalisation[23,24] and work has begun to reduce this risk,[25] focussing on reducing inappropriate prescribing of non-steroidal anti-inflammatory drugs (NSAIDS), proton pump inhibitors (PPIs), and duplicate therapy.[26] However progress is inconsistent and large inter-practice variation in levels of polypharmacy remains.[27]

In response to the issues raised in managing increasing numbers of multimorbid patients there have been calls in the UK for improved integration of existing guidelines[28] with the maxim ‘treat the patient, not the disease’. In response the MITCON project has begun which involves the development of a new computer based tool which will automatically detect conflict between guidelines.[Litchfield I, Bordbar B, Bowles J, et al. A protocol for the development of automated conflict resolution between multiple clinical pathways. 2017. Unpublished.] To ensure that this tool is applicable and relevant we need to understand how multiple guidelines are being translated into existing systems and processes and the extent and implications for patients and care providers of prescribing multiple medications. This protocol details the **Mi**xed **M**ethods **M**ultimorbidity **S**tudy (MiMMS) which seeks to both identify barriers and facilitators to successful guideline implementation, and determine current levels of polypharmacy in multimorbid patients in the West Midlands.

For peer review only

Research Questions

There are two distinct yet related research questions that we will answer. The first concerns the way in which guidelines are used in managing multimorbid patients in two settings – primary and secondary care. The second will involve a quantitative assessment of polypharmacy in multimorbid patients in primary care.

1. Managing multimorbidity

We will use the Theoretical Domains Framework (TDF)[29,30] to identify barriers around managing patients with multimorbidity. This framework has been extensively validated within this setting,[31-36] and allows linkage of behaviour change techniques[37] if required later in the project. We will also use Normalisation Process Theory (NPT)[38] to identify the current successful implementation strategies for guidelines and associated computer based tools used within current practice. This theory has also been extensively validated and can assess the process whereby new items become routine practice. To the best of our knowledge, this work is the first to use a dual framework approach for analysis.

2. Prevalence of multimorbidity

We aim to assess current levels of polypharmacy for up to six chronic conditions within primary care retrospectively over a 24 month period. In doing so, we will determine the percentage of patients that experience polypharmacy and whether there are patients with certain combinations of conditions that are most at risk. The literature indicates that our target conditions will occur in at least a pairwise combination. We will therefore perform pairwise comparisons of conditions to assess numbers of patients who have interactions

For peer review only

within their prescribed medications.[39-53] This will allow us to determine which combinations of conditions are most frequently associated with polypharmacy.

METHODS AND ANALYSIS

Settings and Participants

Site recruitment

We will assess all primary care centres in the West Midlands for study eligibility (defined as using EMIS as their clinical system). We will also purposively sample up to four secondary care centres with differing patient demographics in the West Midlands. Finally, we will purposively sample up to two pharmacy centres.

Research design

We are using a mixed methods approach in this work and will be conducting the study in two interrelated phases. Phase I is the qualitative exploration of the implementation of current guidelines including the barriers and enablers to the management of multimorbid patients in a primary and secondary care settings including the use of clinical software systems. Phase II will use quantitative methods to explore the correlation between clinical condition combinations and the numbers of interacting medications prescribed in primary care.

Phase I

Site Recruitment

In accordance with the theory of maximum variation,[54] between 4 and 8 primary care sites and up to 4 secondary care sites will be recruited from the West Midlands region. This

For peer review only

will also help mitigate the risk of responder bias.[55] Sites will be initiated once all research and development (R&D) approvals have been met, and all necessary documents have been returned to the coordinating centre.

Recruitment for these sites will occur through written expressions of interest via the Primary Care Research Network (PCRN) as well as opportunistic recruitment of General Practioners (GPs) during appropriate educational meetings. We will use purposive sampling for the secondary care and community pharmacy sites and will aim to recruit within different working environments e.g. community pharmacy and pharmacists working within a GP practice, where possible. We will also interview healthcare professionals within different departments in secondary care.

Primary care sites that have EMIS web as their clinical system can participate in both phases of the project. We will aim to recruit healthcare professionals who have used other clinical systems such as TPP (SystmOne) or Vision to identify specific barriers relating to these systems.

Healthcare professionals' recruitment

All health care professionals with experience either of managing patients with multimorbidity, or who have implemented new guidelines locally, will be invited to participate in Phase I. In primary care we expect to recruit fully qualified, and registrar level GPs; advanced nurse practitioners; and practice managers. Within secondary care we will recruit participants from specialities relevant to the 6 diseases targeted in the quantitative work. These will include but not be limited to consultants; senior registrars (at least ST4 and

For peer review only

above); and advanced nurse practitioners. Additionally, we will aim to recruit a minimum of two community pharmacists.

In each case we will aim to recruit at least two individuals from each of the staff groups stated above, and we will continue with purposive sampling until data saturation has been achieved.[56]

Data management

In depth semi structured interviews will be conducted using a topic guide tailored to both the NPT and TDF (Additional file 1) so that both barriers around management of multimorbid patients and current guideline usage can be elicited. Prompts and examples will be used to ensure that data is captured and we will perform constant comparison analysis[57] during the interview process. In depth semi structured interviews will allow these specific topics to be covered, whilst retaining an element of narrative so that healthcare professionals can share the factors that are important to them. Interviewees will always be given the opportunity to add anything they wish at the start and end of the interview. Field notes will be taken by the researcher, both to allow for continued professional development and also to take note of surroundings and reflection of important findings.

Where possible, participants will be sent a copy of the consent form and information sheet at least 24 hours before the interview so that they have time to consider taking part and also time to reflect upon the challenges of managing these types of patients. Using a snowballing technique,[58] sometimes known as the chain referral technique where an

For peer review only

interviewee is asked to suggest a member of their team who may have further insights on a particular topic, this may not always be possible, so if participants require less time to decide to take part they may do so.

Fully informed consent will be taken by a trained researcher who holds a current good clinical practice (GCP) certificate and all associated approvals. We recognise that healthcare professionals may feel uncomfortable sharing cases where they feel that patients may not have been managed to the published guidance, however all participants will be reassured that not all questions have to be answered and the interview can stop at any time. Any data that could identify the individual practice, or patient will be anonymised prior to any form of dissemination. Participants will have up to two weeks after the interview to withdraw their data prior to data analysis.

We will conduct a maximum of 30 interviews, however, interviews will be stopped when data saturation has been reached.[59,60] We will aim to include at least two interviews from each group of healthcare professionals (Consultants; Specialist Registrars; GPs; GP registrars; Practice managers; Advanced nurse practitioners; Community pharmacists). Purposive sampling will be used with snowballing to ensure that the correct mix of healthcare professionals is included.

Data Analysis

All interviews will be recorded using a digital voice recorder and consent for this will be obtained prior to starting. Digital recordings will then be transcribed verbatim prior to analysis.

For peer review only

The returned document will be checked, both for queries and to ensure that anonymity is protected prior to being imported into a suitable analysis program such as NVivo (V11).

TDF will be the main conceptual framework used in the qualitative work. This has been validated in a series of primary and secondary studies[29,31-34,36,61-64] as a method of understanding behaviour within a clinical setting. NPT will also be used as a comparator. This framework has been developed to support the implementation and evaluation of complex interventions[38,65-68] and has been used extensively in trials, systematic reviews and qualitative work.[69] This theory will allow us to assess exactly how a guideline can be successfully used in practice, and how to make a new computer based decision tool to help more effectively manage patients with multimorbidity. Outside of these two frameworks, we will remain sensitive to new themes and will analyse iteratively. We will also perform a comparative analysis between the frameworks if time allows.

Study Outcomes

The outcomes for the qualitative work are:

- To assess how guidelines are being implemented in the management of patients with multimorbidity within primary and secondary care; and
- To understand influences on prescribing for patients with multimorbidity including which factors are taken into consideration and how clinical decisions are made.

Phase II

Site recruitment

For peer review only

Primary care centres who have agreed to take part in phase one and have EMIS as their clinical system will be approached for phase two.

Participant recruitment/data collection

We will use custom built code provided by PRIMIS which generates a report within the GPs' clinical system. The code will be fully tested prior to use to ensure patient anonymity is protected. All eligible patients over the age of 18 who have been registered at the practice for a minimum of three months will have data extracted relating to prescriptions. For patients to be fully eligible they must also have a Read code within their notes at any time for at least one of the target conditions (Table 1) and have had at least one prescription issued within the last two years. All data will remain anonymous and we will extract one possible identifier, sex, to allow us to assess the population demographic. To ensure anonymity, age at the date of extraction will also be collected rather than date of birth. The code will produce three reports; one will detail the practice profile, one will detail the conditions the patient has coded with the Read code included, and the final report will detail the drug history over the preceding 24 months.

Table 1 Read codes

Condition	Included (including exception reporting)	Excluded
Hypertension	G2...<br>G20..%<br>G24.. - G2z.. (Excluding G24z1, G2400, G2410, G27..)<br>Gyu2.<br>Gyu20	21261<br>212K.
COPD	H3...<br>H31..% (excluding H3101, H31y0, H3122)	2126F

For peer review only

	H32..%<br>H36.. - H3z.. (excluding H3y0., H3y1.)<br>H4640<br>H4641<br>H5832<br>Hyu30<br>Hyu31	
Depression	E0013,<br>E0021<br>E112.%,<br>E113.%<br>E118.,<br>E11y2<br>E11z2,<br>E130.<br>E135.<br>E2003<br>E291.,<br>E2B..<br>E2B1.,<br>Eu204<br>Eu251<br>Eu32.% (excluding Eu32A, Eu32B, Eu329) Eu33.%<br>Eu341<br>Eu412	212S.
Diabetes	C10..,<br>C109J,<br>C109K,<br>C10C.,<br>C10D.,<br>C10E.%,<br>C10F.% (Excluding C10F8),<br>C10G.%,<br>C10H.%,<br>C10M.%,<br>C10N.%,<br>PKyP.,<br>C10P.%,<br>C10Q.	21263<br>212H.
Hypertension	G2...<br>G20..%<br>G24.. - G2z.. (Excluding G24z1, G2400, G2410, G27..)<br>Gyu2.<br>Gyu20	21261<br>212K.

For peer review only

Osteoarthritis	N05..00 Osteoarthritis and allied disorders	
CKD	1Z12.<br>1Z13.<br>1Z14.<br>1Z15.<br>1Z16.<br>1Z1B. - 1Z1L.<br>1Z1T.<br>1Z1V.<br>1Z1W.<br>1Z1X.<br>1Z1Y.<br>1Z1Z.<br>1Z1a.<br>1Z1b.<br>1Z1c.<br>1Z1d.<br>1Z1e.<br>1Z1f.<br>K053.<br>K054.<br>K055.<br>1Z10.<br>1Z11.<br>1Z17. - 1Z1A.<br>1Z1M.<br>1Z1Q.<br>K051.<br>K052.<br>1Z1N.<br>1Z1P.<br>1Z1R.<br>1Z1S.	2126E

Sample size

This study uses an audit approach to collect data, therefore there is limited scope for increasing the sample size as there is not a traditional recruitment component. Using the Quality Outcomes Framework (QOF) data from 2014-2015, the average prevalence and list size has been calculated in Table 2. Currently, there is no register of patients with osteoarthritis, so these numbers are not available. Table 3 shows the estimated numbers

For peer review only

extrapolated from Table 2, modelling the effects of practice number. It is likely that these are minimum estimates as patients may have associated Read codes but have not been included on the disease register. Currently we have no data for the number of prescribed drugs that currently interact for this population. Assuming that 10% of drugs prescribed have an interaction, we can assume that a minimum of 53 and a maximum of 806 parameters can be assessed (Table 3) which will be sufficient for the fixed effects model. Therefore in this feasibility study we aim to recruit a minimum of four primary care centres.

Table 2: Estimates within UK primary care between 2014 - 2015

	List size (total number)	List size (estimated 30-74 years)	CHD (Number on register)	Hypertension (Number on register)	COPD (Number on register)	Diabetes Mellitus 17 years and above (Number on register)	Depression (Number on register)
**Average**	7,304	4,049	237	1,007	133	375	425
**Standard Deviation**	4,429	2,446	167	650	98	233	338
**Inter quartile range (IQR)**	5,870	3,323	215	854	119	301	400

Table 3: Minimum and maximum estimates for selected conditions

	4 GP practices	6 GP practices	8 GP practices
Estimated average list	29,216	43,824	58,432
Estimated average list of 30-74	16,196	24,294	32,392
Estimated number of Coronary heart disease patients	948	1,422	1,896
Estimated number of Hypertension patients	4,028	6,042	8,056
Estimated number of COPD patients	532	798	1,064
Estimated number of Diabetes Mellitus patients	1,500	2,250	3,000

For peer review only

Estimated number of Depression patients	1,700	2,550	3,400

Data management

The QOF business rules version 34 will be used to extract Read codes for five of our six target conditions (Chronic Obstructive Pulmonary Disease (COPD), Type 2 Diabetes, Depression, Coronary Heart Disease (CHD), and Hypertension). The general parent code will be used to extract patients suffering from Osteoarthritis as there are currently no QOF indicators that are suitable for this (Table 1). Every patient identified using these codes will have retrospective prescription data collected for the preceding two years. Unique subject identification numbers will be generated so that a comprehensive picture of prescriptions can be built up for each patient, whilst remaining anonymised. The clinical extraction code containing these details will be generated by a company with expertise in this area of work so that only relevant data is extracted and patient anonymity is preserved.

After the patient's drug history has been constructed, we will use our new drug interaction tool to assess the numbers and types of conflicts. This tool is based upon the BNF interactions list and is the current gold standard. This work will help validate the first module within the larger software based that is being developed within the grant. We will also extract data relating to chronic kidney disease (CKD) stage and we will use this data to interpret our findings due to changes in renal function often triggering a change in prescriptions. We recognise that there are challenges managing patients with multimorbidity, and that there may be a time when drugs that interact have to be used due to the clinical severity of one or more conditions. Therefore this data is not going to be

For peer review only

linked to specific prescribers, and data will be fed back to practices in a pseudo-anonymised form.

A practice age demographic profile will be gained from the practice and publicly available QOF records, so that the results can be interpreted in the correct context. These results will also be compared to national records of disease profiling using the QOF disease registers and associated prevalence.

Data analysis

Data will be analysed using a fixed effects model with interaction terms. The interaction term will be whether a drug pair is appropriate or inappropriate as defined using the current gold standard method, with a variable of presence or absence of condition. Pairwise combinations of all conditions will be explored, as the literature suggests that each combination of two diseases has an evidence base.

Study outcomes

The primary outcome for phase two is:

- To elicit the proportion of patients with pairwise condition combinations who have been prescribed two or more drugs and the type of interaction between the two.

Further secondary outcomes for this work are to elicit:

- The total number, and severity, of interactions for each clinical condition as defined by the latest gold standard references.

For peer review only

- To compare the numbers of patients identified with these clinical conditions with the practice disease register and to the national reported prevalence using the latest available QOF data.

DISCUSSION

This study protocol is using a mixed methods approach to better understand the challenges associated with multimorbidity and polypharmacy. In determining the utilisation of guidelines in the management of multiple morbidities we will use two methodological approaches. Both Theoretical Domains Framework[29,31,33-36,63,64,70,71] and Normalisation Process Theory have been rigorously evaluated and used within the National Health Service (NHS) context.[38,72-75]. The dual analysis will enable a more robust understanding of the current issues. By extracting prescribing data on multimorbid patients for the first time we will be able to quantify the prevalence of polypharmacy in patients with combinations of some of the most common conditions.

There are also a number of potential limitations with our approach. Firstly, there is a risk of responder bias at both the practice recruitment and interviewee recruitment stages. Our findings will not necessarily be representative of the whole of the UK however by conducting our study in the West Midlands we have the opportunity to recruit from a range of practices in terms of size and socio-economic environment and staff with a range of seniority and experience. Also, by conducting semi-structured interviews with individual members of staff we expect to reduce the impact of social desirability bias. Finally, the majority of our interviews in primary care are running the clinical system EMIS which may

For peer review only

have different challenges to the other clinical systems currently running. We seek to address this by recruiting at a specialist educational meeting to include at least two GPs running a different clinical system. Also, during the training period, it is likely that GPs will have used other systems, so we will prompt around this area so that this limitation is minimised.

The findings of each phase will be important in their own right but taken together they will provide a rigorous and robust description of the current issues around managing multimorbidity and the prevalence of polypharmacy. This information will prove invaluable in the development of our novel software tool to ensure that it can be tailored to barriers most amenable to change.

ETHICS AND DISSEMINATION

Ethics

Favourable ethical approval has been granted by The University of Birmingham Research Ethics Committee (ERN_16-0074) on 17th May 2016.

Dissemination

Our work will be disseminated through peer reviewed literature, trade journals, and conferences. We will also use the dedicated web page hosted by the University to serve as a central point of contact and as a repository of our findings. We aim to produce a minimum of three articles from this work to contribute to the international scientific literature.

DECLARATIONS

Trial status

For peer review only

At the time of submission, this study was actively recruiting centres and participants and no analysis work has yet been undertaken.

Authors' contributions

RB wrote the first draft of the protocol and all authors participated in further drafting. AMT provided specialist clinical knowledge. PW and ML provided specialist IT knowledge for the analysis of the polypharmacy data. RB and IL conceived the original idea for this study. All authors read and approved the final manuscript.

Competing interests

None declared.

Funding

This work was supported by the Engineering and Physical Sciences Research Council (Grant No. EP/M014401/1).

Data sharing

Not applicable.

Acknowledgements

This work forms part of the grant 'Automated Conflict Resolution in Clinical Pathways' (EP/M014401/1) which has been funded by the Engineering and Physical Sciences Research Council (EPSRC). The custom built code was funded by The University of Birmingham College of Medical and Dental Science Research Development Fund.

For peer review only

Abbreviations

Chronic kidney Disease (CKD); Chronic Obstructive Pulmonary Disease (COPD); Coronary Heart Disease (CHD); General Practioner (GP); Good Clinical Practice (GCP); National Health Service (NHS); National Institute of Clinical Excellence (NICE); Normalisation Process Theory (NPT); Non-Steroidal Anti-inflammatory Drugs (NSAIDS); Proton Pump Inhibitors (PPIs); Quality Outcomes Framework (QOF); Theoretical Domains Framework (TDF); United Kingdom (UK).

REFERENCES

1 Eccles MP, Grimshaw JM, Shekelle P, *et al.* Developing clinical practice guidelines: target audiences, identifying topics for guidelines, guideline group composition and functioning and conflicts of interest. *Implement Sci* 2012;**7**:60. doi:10.1186/1748-5908-7-60

2 Shekelle P, Woolf S, Grimshaw JM, *et al.* Developing clinical practice guidelines: reviewing, reporting, and publishing guidelines; updating guidelines; and the emerging issues of enhancing guideline implementability and accounting for comorbid conditions in guideline development. *Implement Sci* 2012;**7**:62. doi:10.1186/1748-5908-7-62

3 Woolf S, Schünemann HJ, Eccles MP, *et al.* Developing clinical practice guidelines: types of evidence and outcomes; values and economics, synthesis, grading, and presentation and deriving recommendations. *Implement Sci* 2012;**7**:61.

For peer review only

doi:10.1186/1748-5908-7-61

4 Campbell H, Hotchkiss R, Bradshaw N, *et al.* Integrated care pathways. *BMJ* 1998;**316**:133-7. doi:10.1136/bmj.316.7125.133

5 Hawley ST, Zikmund-Fisher B, Ubel P, *et al.* The impact of the format of graphical presentation on health-related knowledge and treatment choices. *Patient Educ Couns* 2008;**73**:448-55. doi:10.1016/j.pec.2008.07.023

6 Diagnostic therapeutic flow-charts for low back pain patients: the Italian clinical guidelines - Europa Medicophysica 2006 June;42(2):151-70 - Minerva Medica - Journals. http://www.minervamedica.it/en/journals/europa-medicophysica/article.php?cod=R33Y2006N02A0151 (accessed 28 Feb 2017).

7 Li W, Liu K, Yang H, *et al.* Integrated clinical pathway management for medical quality improvement – based on a semiotically inspired systems architecture. *Eur J Inf Syst* 2014;**23**:400-17. doi:10.1057/ejis.2013.9

8 Schedlbauer A, Prasad V, Mulvaney C, *et al.* What Evidence Supports the Use of Computerized Alerts and Prompts to Improve Clinicians' Prescribing Behavior? *J Am Med Informatics Assoc* 2009;**16**:531-8. doi:10.1197/jamia.M2910

9 Roshanov PS, Misra S, Gerstein HC, *et al.* Computerized clinical decision support systems for chronic disease management: A decision-maker-researcher partnership systematic review. *Implement Sci* 2011;**6**:92. doi:10.1186/1748-5908-6-92

10 Roshanov PS, Fernandes N, Wilczynski JM, *et al.* Features of effective computerised clinical decision support systems: meta-regression of 162 randomised trials. *BMJ* 2013;**346**:f657. doi:10.1136/bmj.f657

11 Kesselheim AS, Cresswell K, Phansalkar S, *et al.* Clinical decision support systems could be modified to reduce 'alert fatigue' while still minimizing the risk of litigation.

For peer review only

Health Aff (Millwood) 2011;**30**:2310-7. doi:10.1377/hlthaff.2010.1111

12 Published | Guidance | NICE. http://www.nice.org.uk/guidance/published?type=guidelines (accessed 28 Feb 2017).

13 Smith SM, Soubhi H, Fortin M, *et al.* Managing patients with multimorbidity: systematic review of interventions in primary care and community settings. *BMJ* 2012;**345**:e5205-e5205. doi:10.1136/bmj.e5205

14 Long Term Conditions Compendium of Information: Third Edition - Publications - GOV.UK. https://www.gov.uk/government/publications/long-term-conditions-compendium-of-information-third-edition (accessed 28 Feb 2017).

15 Dumbreck S, Flynn A, Nairn M, *et al.* Drug-disease and drug-drug interactions: systematic examination of recommendations in 12 UK national clinical guidelines. *BMJ* 2015;**350**:h949. doi:10.1136/bmj.h949

16 Hughes LD, McMurdo MET, Guthrie B. Guidelines for people not for diseases: the challenges of applying UK clinical guidelines to people with multimorbidity. *Age Ageing* 2013;**42**:62-9. doi:10.1093/ageing/afs100

17 Guthrie B, Payne K, Alderson P, *et al.* Adapting clinical guidelines to take account of multimorbidity. *BMJ* 2012;**345**:e6341. doi:10.1136/bmj.e6341

18 Fried TR, Tinetti ME, Iannone L. Primary Care Clinicians' Experiences With Treatment Decision Making for Older Persons With Multiple Conditions. *Arch Intern Med* 2010;**171**:75-80. doi:10.1001/archinternmed.2010.318

19 Polypharmacy and medicines optimisation. http://www.volgmed.ru/uploads/files/2014-12/35099-prilozhenie_1-3_k_informacionnomu_pismu_2.pdf (accessed 28 Feb 2017).

20 Guthrie B, McCowan C, Davey P, *et al.* High risk prescribing in primary care patients

For peer review only

particularly vulnerable to adverse drug events: cross sectional population database analysis in Scottish general practice. *BMJ* 2011;**342**:d3514. doi:10.1136/bmj.d3514

21 Sinnott C, Bradley C, Bradley CP. Multimorbidity or polypharmacy: two sides of the same coin? *J Comorbidity* 2015;**5**:29-31. doi:10.15256/joc.2015.5.51

22 Sinnott C, Hugh SM, Boyce MB, *et al.* What to give the patient who has everything? A qualitative study of prescribing for multimorbidity in primary care. *Br J Gen Pract* 2015;**65**:e184-91. doi:10.3399/bjgp15X684001

23 Payne RA, Abel GA, Avery AJ, *et al.* Is polypharmacy always hazardous? A retrospective cohort analysis using linked electronic health records from primary and secondary care. *Br J Clin Pharmacol* 2014;**77**:1073-82. doi:10.1111/bcp.12292

24 Cahir C, Bennett K, Teljeur C, *et al.* Potentially inappropriate prescribing and adverse health outcomes in community dwelling older patients. *Br J Clin Pharmacol* 2014;**77**:201-10. doi:10.1111/bcp.12161

25 Bemben NM. Deprescribing: An Application to Medication Management in Older Adults. *Pharmacother J Hum Pharmacol Drug Ther* 2016;**36**:774-80. doi:10.1002/phar.1776

26 Twigg MJ, Wright D, Barton GR, *et al.* The four or more medicines (FOMM) support service: results from an evaluation of a new community pharmacy service aimed at over-65s. *Int J Pharm Pract* 2015;**23**:407-14. doi:10.1111/ijpp.12196

27 Sinnige J, Braspenning JC, Schellevis FG, *et al.* Inter-practice variation in polypharmacy prevalence amongst older patients in primary care. *Pharmacoepidemiol Drug Saf* 2016;**25**:1033-41. doi:10.1002/pds.4016

28 Kenning C, Fisher L, Bee P, *et al.* Primary care practitioner and patient understanding of the concepts of multimorbidity and self-management: A qualitative study. *SAGE*

For peer review only

open Med 2013;**1**:2050312113510001. doi:10.1177/2050312113510001

29 Cane J, O'Connor D, Michie S. Validation of the theoretical domains framework for use in behaviour change and implementation research. *Implement Sci* 2012;**7**:37. doi:10.1186/1748-5908-7-37

30 Michie S, Johnston M, Francis J, *et al.* From Theory to Intervention: Mapping Theoretically Derived Behavioural Determinants to Behaviour Change Techniques. *Appl Psychol* 2008;**57**:660-80. doi:10.1111/j.1464-0597.2008.00341.x

31 Patey AM, Islam R, Francis JJ, *et al.* Anesthesiologists' and surgeons' perceptions about routine pre-operative testing in low-risk patients: application of the Theoretical Domains Framework (TDF) to identify factors that influence physicians' decisions to order pre-operative tests. *Implement Sci* 2012;**7**:52. doi:10.1186/1748-5908-7-52

32 Curran JA, Brehaut J, Patey AM, *et al.* Understanding the Canadian adult CT head rule trial: use of the theoretical domains framework for process evaluation. *Implement Sci* 2013;**8**:25. doi:10.1186/1748-5908-8-25

33 Duncan EM, Francis JJ, Johnston M, *et al.* Learning curves, taking instructions, and patient safety: using a theoretical domains framework in an interview study to investigate prescribing errors among trainee doctors. *Implement Sci* 2012;**7**:86. doi:10.1186/1748-5908-7-86

34 French SD, Green SE, O'Connor DA, *et al.* Developing theory-informed behaviour change interventions to implement evidence into practice: a systematic approach using the Theoretical Domains Framework. *Implement Sci* 2012;**7**:38. doi:10.1186/1748-5908-7-38

35 Islam R, Tinmouth AT, Francis JJ, *et al.* A cross-country comparison of intensive care physicians' beliefs about their transfusion behaviour: a qualitative study using the

For peer review only

Theoretical Domains Framework. *Implement Sci* 2012;**7**:93. doi:10.1186/1748-5908-7-93

36 Bussières AE, Patey AM, Francis JJ, *et al.* Identifying factors likely to influence compliance with diagnostic imaging guideline recommendations for spine disorders among chiropractors in North America: a focus group study using the Theoretical Domains Framework. *Implement Sci* 2012;**7**:82. doi:10.1186/1748-5908-7-82

37 Michie S, Richardson M, Johnston M, *et al.* The behavior change technique taxonomy (v1) of 93 hierarchically clustered techniques: building an international consensus for the reporting of behavior change interventions. *Ann Behav Med* 2013;**46**:81-95. doi:10.1007/s12160-013-9486-6

38 May C, Finch T. Implementing, Embedding, and Integrating Practices: An Outline of Normalization Process Theory. *Sociology* 2009;**43**:535-54. doi:10.1177/0038038509103208

39 Stage KB, Middelboe T, Stage TB, *et al.* Depression in COPD – management and quality of life considerations. *Int J Chron Obstruct Pulmon Dis* 2006;**1**:315-20. http://www.pubmedcentral.nih.gov/articlerender.fcgi?artid=2707161&tool=pmcentrez&rendertype=abstract (accessed 28 Feb 2017).

40 Rana JS, Mittleman MA, Sheikh J, *et al.* Chronic Obstructive Pulmonary Disease, Asthma, and Risk of Type 2 Diabetes in Women. *Diabetes Care* 2004;**27**:2478-84. doi:10.2337/diacare.27.10.2478

41 Lee TA, Pickard AS, Bartle B, *et al.* Osteoarthritis: a comorbid marker for longer life? *Ann Epidemiol* 2007;**17**:380-4. doi:10.1016/j.annepidem.2007.01.033

42 Chaouat A, Naeije R, Weitzenblum E. Pulmonary hypertension in COPD. *Eur Respir J* 2008;**32**:1371-85. doi:10.1183/09031936.00015608

For peer review only

43 Campo G, Pavasini R, Malagù M, *et al.* Chronic obstructive pulmonary disease and ischemic heart disease comorbidity: overview of mechanisms and clinical management. *Cardiovasc Drugs Ther* 2015;**29**:147-57. doi:10.1007/s10557-014-6569-y

44 Mezuk B, Eaton WW, Albrecht S, *et al.* Depression and type 2 diabetes over the lifespan: a meta-analysis. *Diabetes Care* 2008;**31**:2383-90. doi:10.2337/dc08-0985

45 Lin EHB. Depression and osteoarthritis. *Am J Med* 2008;**121**:S16-9. doi:10.1016/j.amjmed.2008.09.009

46 Rubio-Guerra AF, Rodriguez-Lopez L, Vargas-Ayala G, *et al.* Depression increases the risk for uncontrolled hypertension. *Exp Clin Cardiol* 2013;**18**:10-2. http://www.pubmedcentral.nih.gov/articlerender.fcgi?artid=3716493&tool=pmcentrez&rendertype=abstract (accessed 28 Feb 2017).

47 Rivelli S, Jiang W. Depression and ischemic heart disease: what have we learned from clinical trials? *Curr Opin Cardiol* 2007;**22**:286-91. doi:10.1097/HCO.0b013e3281ead011

48 Berenbaum F. Diabetes-induced osteoarthritis: from a new paradigm to a new phenotype. *Postgrad Med J* 2012;**88**:240-2. doi:10.1136/pgmj.2010.146399rep

49 Cheung BMY, Li C. Diabetes and hypertension: is there a common metabolic pathway? *Curr Atheroscler Rep* 2012;**14**:160-6. doi:10.1007/s11883-012-0227-2

50 Juutilainen A, Lehto S, Ronnemaa T, *et al.* Type 2 Diabetes as a 'Coronary Heart Disease Equivalent': An 18-year prospective population-based study in Finnish subjects. *Diabetes Care* 2005;**28**:2901-7. doi:10.2337/diacare.28.12.2901

51 Mackenzie IS, MacDonald TM. Treatment of osteoarthritis in hypertensive patients. *Expert Opin Pharmacother* 2010;**11**:393-403. doi:10.1517/14656560903496422

52 Rahman MM, Kopec JA, Cibere J, *et al.* The relationship between osteoarthritis and cardiovascular disease in a population health survey: a cross-sectional study. *BMJ Open* 2013;**3**. doi:10.1136/bmjopen-2013-002624

53 Špinar J. Hypertension and ischemic heart disease. *Cor Vasa* 2012;**54**:e433-8. doi:10.1016/j.crvasa.2012.11.002

54 Palinkas LA, Horwitz SM, Green CA, *et al.* Purposeful Sampling for Qualitative Data Collection and Analysis in Mixed Method Implementation Research. *Adm Policy Ment Health* 2015;**42**:533-44. doi:10.1007/s10488-013-0528-y

55 Paulhus DL, L. D. Measurement and Control of Response Bias. In: *Measures of Personality and Social Psychological Attitudes*. Elsevier 1991. 17-59. doi:10.1016/B978-0-12-590241-0.50006-X

56 Mason M. Sample Size and Saturation in PhD Studies Using Qualitative Interviews. *Forum Qual Sozialforsch / Forum Qual Soc Res* 2010;**11**.

57 Glaser BG. The Constant Comparative Method of Qualitative Analysis. *Soc Probl* 1965;**12**(4):436-445. http://www.jstor.org/stable/798843?seq=1#page_scan_tab_contents (accessed 28 Feb 2017).

58 Berg S. Snowball Sampling-I. In: *Encyclopedia of Statistical Sciences*. John Wiley & Sons, Inc. 2004. doi:10.1002/0471667196.ess2478.pub2

59 Green J, Thorogood N. *Qualitative Methods for Health Research*. SAGE Publications 2013. https://books.google.com/books?hl=en&lr=&id=qg0tAgAAQBAJ&pgis=1 (accessed 28 Feb 2017).

60 *Qualitative Researching with Text, Image and Sound: A Practical Handbook for Social Research*. SAGE Publications 2000.

For peer review only

https://books.google.com/books?hl=en&lr=&id=Ab9Z1Js2LDoC&pgis=1 (accessed 28 Feb 2017).

61 Tavender EJ, Bosch M, Gruen RL, *et al.* Understanding practice: the factors that influence management of mild traumatic brain injury in the emergency department--a qualitative study using the Theoretical Domains Framework. *Implement Sci* 2014;**9**:8. doi:10.1186/1748-5908-9-8

62 Islam R, Tinmouth AT, Francis JJ, *et al.* A cross-country comparison of intensive care physicians' beliefs about their transfusion behaviour: a qualitative study using the Theoretical Domains Framework. *Implement Sci* 2012;**7**:93. doi:10.1186/1748-5908-7-93

63 Boscart VM, Fernie GR, Lee JH, *et al.* Using psychological theory to inform methods to optimize the implementation of a hand hygiene intervention. *Implement Sci* 2012;**7**:77. doi:10.1186/1748-5908-7-77

64 Francis JJ, O'Connor D, Curran J. Theories of behaviour change synthesised into a set of theoretical groupings: introducing a thematic series on the theoretical domains framework. *Implement Sci* 2012;**7**:35. doi:10.1186/1748-5908-7-35

65 Finch T. Teledermatology for chronic disease management: coherence and normalization. *Chronic Illn* 2008;**4**:127-34. doi:10.1177/1742395308092483

66 May C. Towards a general theory of implementation. *Implement Sci* 2013;**8**:18. doi:10.1186/1748-5908-8-18

67 *The New Sociology of the Health Service*. Routledge 2009. https://books.google.com/books?id=Mwb3GeIFr4oC&pgis=1 (accessed 28 Feb 2017).

68 Pescosolido BA, Martin JK, McLeod JD, *et al.*, editors. *Handbook of the Sociology of Health, Illness, and Healing*. New York, NY: Springer New York 2011. doi:10.1007/978-

For peer review only

1-4419-7261-3

69 NPT Bibliography. http://www.normalizationprocess.org/bibliography/ (accessed 28 Feb 2017).

70 Tavender EJ, Bosch M, Gruen RL, *et al.* Understanding practice: the factors that influence management of mild traumatic brain injury in the emergency department--a qualitative study using the Theoretical Domains Framework. *Implement Sci* 2014;**9**:8. doi:10.1186/1748-5908-9-8

71 Curran JA, Brehaut J, Patey AM, *et al.* Understanding the Canadian adult CT head rule trial: use of the theoretical domains framework for process evaluation. *Implement Sci* 2013;**8**:25. doi:10.1186/1748-5908-8-25

72 Bamford C, Heaven B, May C, *et al.* Implementing nutrition guidelines for older people in residential care homes: a qualitative study using Normalization Process Theory. *Implement Sci* 2012;**7**:106. doi:10.1186/1748-5908-7-106

73 Bouamrane M-M, Mair FS, Edwards N, *et al.* A qualitative evaluation of general practitioners' views on protocol-driven eReferral in Scotland. *BMC Med Inform Decis Mak* 2014;**14**:30. doi:10.1186/1472-6947-14-30

74 Franx G, Oud M, de Lange J, *et al.* Implementing a stepped-care approach in primary care: results of a qualitative study. *Implement Sci* 2012;**7**:8. doi:10.1186/1748-5908-7-8

75 Lhussier M, Forster N, Eaton S, *et al.* Care planning for long term conditions in primary care: indicators of embeddedness. *Eur J Pers Centered Healthc* 2015;**3**:59. doi:10.5750/ejpch.v3i1.869

For peer review only

Additional file 1: Topic guide for GPs

Question	NPT construct	TDF domain
• What challenges do you face as a GP treating patients with MM?<br>○ How confident do you feel managing a complex patient?<br>○ Which tools help you to manage these patients?<br>○ What is it about these tools that make them effective?	• Differentiation between tools<br>• Communal specification	• Social/professional role<br>• Beliefs about capabilities<br>• Memory, attention and decision processes<br>• Environmental context and resources<br>• Behavioural regulation
• Are there any specific challenges with COPD and OA patients? (and other 5 conditions)<br>○ Context or systems?<br>○ What makes it hard or easy<br>○ If you could change one thing to make it easier to manage these patients, what would it be<br>○ What advice would you give to a GP registrar to help them manage patients with MM?	• Internalisation	• Knowledge<br>• Environmental context<br>• Emotion<br>• Beliefs about capabilities
• Have you worked in any practice that had a different, (more helpful) way of managing these patients?<br>○ Can you give an example…	• Legitimation<br>• Skill set workability<br>• Systematization<br>• Activation	• Environmental context<br>• Social influences?
• How are guidelines implemented within your practice?<br>○ Is there one specific person in the practice that is responsible for this?<br>○ Are there lots of local audits performed and fed back? If so, have you seen any changes from these?	• Initiation<br>• Enrolment<br>• Interactional workability<br>• Skill set workability<br>• Contextual integration<br>• Communal appraisal	• Social/professional role<br>• Environmental context<br>• Reinforcement<br>• Goals
• How do you use guidelines within your day to day practice	• Contextual integration	• Knowledge<br>• Skills

For peer review only

		• Memory
• Have you ever seen any successful changes in practice following new guidelines being used? (looking for successful implementation strategies)<br>○ What made you have confidence in this system?	• Systematization<br>• Relational integration	• Optimism<br>• Goals<br>• Social influences
• Could you tell me about your current practice clinical system and any barriers you have found it presents to your efficiency within a clinic?<br>○ Have you used other systems and found this to be a consistent problem throughout?	• Reconfiguration<br>• Individual appraisal	• Environment<br>• Emotion<br>• Knowledge<br>• Optimism
• We are making a new tool with automated prompts, how does that make you feel?<br>○ What would make a tool like this useful?	• Individual appraisal	• Beliefs about consequences<br>• Intentions<br>• Knowledge<br>• Emotion
• When a patient requires treating with two drugs that are not advised to be used together, can you tell me what factors you use to resolve this?<br>○ How do you choose one drug over another?<br>○ Has litigation shaped your practice?		• Knowledge<br>• Skill<br>• Beliefs about consequences<br>• Beliefs about capabilities<br>• Social influences

For peer review only

BMJ Open

Assessing the extent of drug interactions amongst patients with multimorbidity in primary and secondary care in the West Midlands: A study protocol for the Mixed Methods Multimorbidity Study (MiMMS)

Journal:	*BMJ Open*
Manuscript ID	bmjopen-2017-016713.R1
Article Type:	Protocol
Date Submitted by the Author:	02-Jun-2017
Complete List of Authors:	Backman, Ruth; University of Birmingham, Institute of Applied Health Research<br>Weber, Philip; University of Birmingham, School of Computer Science<br>Turner, Alice; University of Birmingham, Institute of Inflammation & Aging; Heart of England NHS Foundation Trust<br>Lee, Mark; University of Birmingham, School of Computer Science<br>Litchfield, Ian; University of Birmingham, Institute of Applied Health Research
**Primary Subject Heading**:	Medical management
Secondary Subject Heading:	Evidence based practice, Qualitative research, Pharmacology and therapeutics
Keywords:	Protocol, Multimorbidity, Polypharmacy, NHS, PRIMARY CARE, SECONDARY CARE

SCHOLARONE™
Manuscripts

Assessing the extent of drug interactions amongst patients with multimorbidity in primary and secondary care in the West Midlands: A study protocol for the Mixed Methods Multimorbidity Study (MiMMS)

For peer review only

Ruth Backman[1], Philip Weber[2], Alice M Turner[3,4], Mark Lee[2], Ian Litchfield[1‡].

[1] Institute of Applied Health Research, University of Birmingham, Edgbaston, Birmingham B15 2TT

[2] School of Computer Science, University of Birmingham, Edgbaston, Birmingham B15 2TT

[3] Heartlands Hospital, Heart of England NHS Foundation Trust, Bordesley Green East, Birmingham B9 5SS

[4] Institute of Inflammation & Aging, University of Birmingham, Edgbaston, Birmingham B15 2WB

[‡] Corresponding author

Address: Institute of Applied Health Research, College of Medical and Dental Sciences
University of Birmingham, Edgbaston, Birmingham B15 2TT
United Kingdom

E-mail: i.litchfield@bham.ac.uk

Tel: +44 (0)121 414 6006

Word count: 3579

Word count (Abstract): 288

ABSTRACT

Introduction: The numbers of patients with three or more chronic conditions (multimorbidity) are increasing, and will rise to 2.9 million by 2018 in the United Kingdom alone. Currently in the UK, conditions are mainly managed using over 250 sets of single condition guidance, which has the potential to generate conflicting recommendations for lifestyle and concurrent medication for individual patients with more than one condition. To address some of these issues, we are developing a new computer-based tool to help manage these patients more effectively. For this tool to be applicable and relevant to current practice we must first better understand how existing patients with multimorbidity are being managed, particularly relating to concerns over prescribing and potential polypharmacy.

Methods and analysis: Up to four secondary care centres, two community pharmacies and between four to eight primary care centres in the West Midlands will be recruited. Interviewees will be purposively sampled from these sites, up to a maximum of 30. In this mixed methods study (MiMMS), we will perform a dual framework analysis on the qualitative data; the first analysis will use the Theoretical Domains Framework to assess barriers and enablers for healthcare professionals around the management of multimorbid patients; the second analysis will use Normalisation Process Theory to understand how interventions are currently being successfully implemented in both settings. We will also extract quantitative anonymised patient data from primary care to determine the extent of polypharmacy currently present for patients with multimorbidity in the West Midlands.

For peer review only

Discussion: We aim to combine these data so that we can build a useful, fully implementable tool which addresses the barriers most amenable to change within both primary and secondary care contexts.

Protocol registration: NIHR Clinical Research Network Portfolio Registration CPMS ID 30613.

Strengths and limitations of this study:

- We are using a series of semi-structured interviews to elicit current barriers found within both primary and secondary care when managing patients with multimorbidity.
- This will be complement by the first quantitative exploration of polypharmacy in multimorbid patients in primary care by interrogating routinely collected prescribing data within the same practices.
- This pilot study is based in the West Midlands whose diverse population mean that a range of practices and clinicians will be participating.

Keywords: Protocol; Multimorbidity; Polypharmacy; NHS; Primary care; Secondary care.

INTRODUCTION

In the United Kingdom (UK) there is a focus on the formation and implementation of guidelines to help standardise and improve the quality of care.[1–3] Providers may choose to translate this guidance into local policy to help implementation, for example, a multidisciplinary care plan which details essential steps in the management of patients with

For peer review only

a specific clinical problem.[4] These pathways frequently use graphical descriptions of evidence and options and are typically represented in a single, or series of, flow charts[5,6] and have been shown to have the possibility to improve care if they are implemented correctly.[7] These systems will contain prompts or reminders which in isolation can help to improve practice[8–10] but in combination can lead to alert fatigue.[11]

Of the 250 clinical guidelines published by the National Institute for Clinical Excellence (NICE),[12] only four account for more than one condition. This presents issues for clinicians attempting to use this guidance to manage multimorbidity. Defined as two or more chronic conditions in the same individual,[13] patients with multimorbidity are becoming more prevalent and it is estimated that by 2018 the number of people in the UK with three or more long term conditions will have grown to 2.9 million.[14] Using multiple single conditions guidelines means patients can be left with a significant treatment burden[15,16] and conflicting advice around lifestyle factors.[17] There are also implications for clinicians who struggle to balance the requirements of several guidelines for multimorbid patients, hindered by the lack of outcome data.[18]

One significant consequence of following multiple guidelines is the concurrent prescribing of multiple medications. This phenomenon has been termed "polypharmacy"[19] and has been identified as a complicating issue for patients with multimorbidity.[20] The chances of polypharmacy greatly increase when several sets of guidance are being implemented in patients with multimorbidity[21] and healthcare professionals recognise that fulfilling multiple guidelines might compromise patient centred care.[22] There is evidence this simultaneous medication administration can lead to an increased risk of patient

For peer review only

hospitalisation[23,24] and work has begun to reduce this risk,[25] focussing on reducing inappropriate prescribing of non-steroidal anti-inflammatory drugs (NSAIDS), proton pump inhibitors (PPIs), and duplicate therapy.[26] However progress is inconsistent and large inter-practice variation in levels of polypharmacy remains.[27]

In response to the issues raised in managing increasing numbers of multimorbid patients there have been calls in the UK for improved integration of existing guidelines[28] with the maxim 'treat the patient, not the disease'. In response the MITCON project has begun which involves the development of a new computer based tool which will automatically detect conflict between guidelines.[Litchfield I, Bordbar B, Bowles J, et al. A protocol for the development of automated conflict resolution between multiple clinical pathways. 2017. Unpublished] To ensure that this tool is applicable and relevant we need to understand how multiple guidelines are being translated into existing systems and processes and the extent and implications for patients and care providers of prescribing multiple medications. This protocol details the **Mi**xed **M**ethods **M**ultimorbidity **S**tudy (MiMMS) which seeks to both identify barriers and facilitators to successful guideline implementation, and determine current levels of polypharmacy in multimorbid patients in the West Midlands.

Research Questions

There are two distinct yet related research questions that we will answer. The first concerns the way in which guidelines are used in managing multimorbid patients in two settings – primary and secondary care. The second will involve a quantitative assessment of polypharmacy in multimorbid patients in primary care. Although previous work has explored multimorbidity, ours is the first to look at both primary and secondary care settings

For peer review only

and corroborate qualitative work with quantitative data on polypharmacy at the same GP practices.

1. Managing multimorbidity

We will use the Theoretical Domains Framework (TDF)[29,30] to identify barriers around managing patients with multimorbidity. This framework has been extensively validated within this setting,[31–36] and allows linkage of behaviour change techniques[30,37] if required later in the project. We will also use Normalisation Process Theory (NPT)[38] to identify the current successful implementation strategies for guidelines and associated computer based tools used within current practice. This theory has also been extensively validated and can assess the process whereby new items become routine practice. To the best of our knowledge, this work is the first to use a dual framework approach for analysis. Furthermore, this work will seek to identify barriers in multiple NHS settings so that the full patient pathway with a number of healthcare professionals can be explored.

2. Prevalence of multimorbidity

We aim to assess current levels of polypharmacy for up to six chronic conditions within primary care retrospectively over a 24-month period. In doing so, we will determine the percentage of patients that experience polypharmacy and whether there are patients with certain combinations of conditions that are most at risk. The literature indicates that our target conditions will occur in at least a pairwise combination. We will therefore perform pairwise comparisons of conditions to assess numbers of patients who have interactions within their prescribed medications.[39–53] This will allow us to determine which combinations of conditions are most frequently associated with polypharmacy. For this

For peer review only

work we are defining multimorbidity as having two or more of our target conditions diagnosed with an active Read code. We are defining polypharmacy as taking a minimum of two drugs at any one time point so that we can assess the interaction.

METHODS AND ANALYSIS

Settings and Participants

Site recruitment

We will assess all primary care centres in the West Midlands for study eligibility (defined as using EMIS as their clinical system). We will also purposively sample up to four secondary care centres with differing patient demographics in the West Midlands. Finally, we will purposively sample up to two pharmacy centres.

Research design

We are using a mixed methods approach in this work and will be conducting the study in two interrelated phases (Figure 1). Phase I is the qualitative exploration of the implementation of current guidelines including the barriers and enablers to the management of multimorbid patients in a primary and secondary care settings including the use of clinical software systems. Phase II will use quantitative methods to explore the correlation between clinical condition combinations and the numbers of interacting medications prescribed in primary care.

Figure 1: Flow diagram of MiMMS

Phase I

For peer review only

Site Recruitment

In accordance with the theory of maximum variation,[54] between 4 and 8 primary care sites and up to 4 secondary care sites will be recruited for this pilot from the West Midlands region. This will also help mitigate the risk of responder bias.[55] Sites will be initiated once all research and development (R&D) approvals have been met, and all necessary documents have been returned to the coordinating centre.

Recruitment for these sites will occur through written expressions of interest via the Primary Care Research Network (PCRN) as well as opportunistic recruitment of General Practioners (GPs) during appropriate educational meetings. We will use purposive sampling for the secondary care and community pharmacy sites and will aim to recruit within different working environments e.g. community pharmacy and pharmacists working within a GP practice, where possible. We will also interview healthcare professionals within different departments in secondary care.

Primary care sites that have EMIS web as their clinical system can participate in both phases of the project. We will aim to recruit healthcare professionals who have used other clinical systems such as TPP (SystmOne) or Vision to identify specific barriers relating to these systems.

Healthcare professionals' recruitment

All health care professionals with experience either of managing patients with multimorbidity, or who have implemented new guidelines locally, will be invited to participate in Phase I. In primary care we expect to recruit fully qualified, and registrar level

For peer review only

GPs; advanced nurse practitioners; and practice managers. Within secondary care we will recruit participants from specialities relevant to the 6 diseases targeted in the quantitative work as well as elderly care physicians if possible. These will include but not be limited to consultants; senior registrars (at least ST4 and above); and advanced nurse practitioners. Additionally, we will aim to recruit a minimum of two community pharmacists. All sampling will be performed using an iterative snowballing approach.[56]

In each case we will aim to recruit at least two individuals from each of the staff groups stated above, and we will continue with purposive sampling until data saturation has been achieved.[57]

Data management

In-depth semi structured interviews will be conducted using a topic guide tailored to both the NPT and TDF (Additional file 1) so that both barriers around management of multimorbid patients and current guideline usage can be elicited. Prompts and examples will be used to ensure that data is captured and we will perform constant comparison analysis[58] during the interview process. In-depth semi structured interviews will allow these specific topics to be covered, whilst retaining an element of narrative so that healthcare professionals can share the factors that are important to them. Interviewees will always be given the opportunity to add anything they wish at the start and end of the interview. Field notes will be taken by the researcher, both to allow for continued professional development and also to take note of surroundings and reflection of important findings.

For peer review only

Where possible, participants will be sent a copy of the consent form and information sheet at least 24 hours before the interview so that they have time to consider taking part and also time to reflect upon the challenges of managing these types of patients. Using a snowballing technique,[56] sometimes known as the chain referral technique where an interviewee is asked to suggest a member of their team who may have further insights on a particular topic, this may not always be possible, so if participants require less time to decide to take part they may do so.

Fully informed consent will be taken by a trained researcher who holds a current good clinical practice (GCP) certificate and all associated approvals. We recognise that healthcare professionals may feel uncomfortable sharing cases where they feel that patients may not have been managed to the published guidance, however all participants will be reassured that not all questions have to be answered and the interview can stop at any time. Any data that could identify the individual practice, or patient will be anonymised prior to any form of dissemination. Participants will have up to two weeks after the interview to withdraw their data prior to data analysis.

We will conduct a maximum of 30 interviews, however, interviews will be stopped when data saturation has been reached and no new major themes are arising within the last five transcripts.[59,60] Due to our interview topics being around a specific aspect of care, combined with interviewees having a similar role and training background we should achieve saturation within our recruitment target.[61] We will aim to include at least two interviews from each group of healthcare professionals (Consultants; Specialist Registrars; GPs; GP registrars; Practice managers; Advanced nurse practitioners; Community

For peer review only

pharmacists). Purposive sampling will be used with snowballing to ensure that the correct mix of healthcare professionals is included.

Data Analysis

All interviews will be recorded using a digital voice recorder and consent for this will be obtained prior to starting. Digital recordings will then be transcribed verbatim prior to analysis.

The returned document will be checked, both for queries and to ensure that anonymity is protected prior to being imported into a suitable analysis program such as NVivo (V11).

TDF will be the main conceptual framework used in the qualitative work. This has been validated in a series of primary and secondary studies[29,31–36,62,63] as a method of understanding behaviour within a clinical setting. NPT will also be used and this framework has been developed to support the implementation and evaluation of complex interventions[38,64,65] and has been used extensively in trials, systematic reviews and qualitative work.[66] This theory will allow us to assess the 'key ingredients' when a guideline or local initiative has been successfully implemented in practice. This information will help us to include ingredients for successful implementation of our new tool to help more effectively manage patients with multimorbidity. Outside of these two frameworks, we will remain sensitive to new themes and will analyse iteratively.

Study Outcomes

For peer review only

The outcomes for the qualitative work are:

- To assess how guidelines and other local initiatives are being implemented in the management of patients with multimorbidity within primary and secondary care with the outcome of defining 'key ingredients' for successful implementation; and
- To understand the barriers and enablers around managing multimorbidity, including prescribing including which factors are taken into consideration and how clinical decisions are made.

Phase II

Site recruitment

Primary care centres who have agreed to take part in phase one and have EMIS as their clinical system will be approached for phase two.

Participant recruitment/data collection

We will use custom built code provided by PRIMIS (The University of Nottingham, 15th Floor Tower Building, University Park, Nottingham. NG7 2RD https://www.nottingham.ac.uk/primis/index.aspx) which generates a report within the GPs' clinical system. The code will be fully tested prior to use to ensure patient anonymity is protected. All eligible patients over the age of 18 who have been registered at the practice for a minimum of three months will have data extracted relating to prescriptions. For patients to be fully eligible they must also have a Read code within their notes at any time for at least one of the target conditions (Supplementary file 1) and have had at least one prescription issued within the last two years. All data will remain anonymous and we will extract one possible identifier, sex, to allow us to assess the population demographic. To

For peer review only

ensure anonymity, age at the date of extraction will also be collected rather than date of birth. The code will produce three reports; one will detail the practice profile, one will detail the conditions the patient has coded with the Read code included, and the final report will detail the drug history over the preceding 24 months.

Sample size

This study uses a retrospective audit approach to collect data, therefore there is limited scope for increasing the sample size as there is not a traditional recruitment component. Using the Quality Outcomes Framework (QOF) data from 2014-2015, the average prevalence and list size has been calculated in Table 1. Currently, there is no register of patients with osteoarthritis, so these numbers are not available. Table 2 shows the estimated numbers extrapolated from Table 1, modelling the effects of practice number. It is likely that these are minimum estimates as patients may have associated Read codes but have not been included on the disease register. Currently we have no data for the number of prescribed drugs that currently interact for this population. Assuming that 10% of drugs prescribed have an interaction, we can assume that a minimum of 53 and a maximum of 806 parameters can be assessed (Table 2) which will be sufficient for the fixed effects model. Therefore in this pilot study we aim to recruit a minimum of four primary care centres up to a maximum of eight.

Table 1: Estimates within UK primary care between 2014 - 2015

	List size (total number)	List size (estimated 30-74 years)	CHD (Number on register)	Hypertension (Number on register)	COPD (Number on register)	Diabetes Mellitus 17 years and above	Depression (Number on register)

For peer review only

						(Number on register)	
**Average**	7,304	4,049	237	1,007	133	375	425
**Standard Deviation**	4,429	2,446	167	650	98	233	338
**Inter quartile range (IQR)**	5,870	3,323	215	854	119	301	400

Table 2: Minimum and maximum estimates for selected conditions

	4 GP practices	6 GP practices	8 GP practices
Estimated average list	29,216	43,824	58,432
Estimated average list of 30-74	16,196	24,294	32,392
Estimated number of Coronary heart disease patients	948	1,422	1,896
Estimated number of Hypertension patients	4,028	6,042	8,056
Estimated number of COPD patients	532	798	1,064
Estimated number of Diabetes Mellitus patients	1,500	2,250	3,000
Estimated number of Depression patients	1,700	2,550	3,400

Data management

The QOF business rules version 34 will be used to extract Read codes for five of our six target conditions (Chronic Obstructive Pulmonary Disease (COPD), Type 2 Diabetes, Depression, Coronary Heart Disease (CHD), and Hypertension). The general parent code will be used to extract patients suffering from Osteoarthritis as there are currently no QOF indicators that are suitable for this (supplementary file 1). Every patient identified using these codes will have retrospective prescription data collected for the preceding two years. Unique subject identification numbers will be generated so that a comprehensive picture of prescriptions can be built up for each patient, whilst remaining anonymised. The clinical

For peer review only

extraction code containing these details will be generated by a company with expertise in this area of work so that only relevant data is extracted and patient anonymity is preserved. We will not use a condition clustering approach and will only use the combinations of conditions as a way to characterise our population rather than drawing conclusions about prevalence of comorbidities.

After the patient's drug history has been constructed using a process mining approach, we will use our new drug interaction tool to assess the numbers and types of conflicts. This tool is based upon the BNF interactions list, so we will use the same categories of no interaction, mild interaction and severe interaction. We will also use the expertise of ML to enable to use of Natural Language Processing within this data set to pull out the types of interaction, and will use any relating to drug clearance to reinterpret those patients with chronic kidney disease. This work will help validate the first module within the larger software based that is being developed within the grant. We will also extract data relating to chronic kidney disease (CKD) stage and we will use this data to interpret our findings due to changes in renal function often triggering a change in prescriptions. We recognise that there are challenges managing patients with multimorbidity, and that there may be a time when drugs that interact have to be used due to the clinical severity of one or more conditions. Therefore this data is not going to be linked to specific prescribers, and data will be fed back to practices in a pseudo-anonymised form.

A practice age demographic profile will be gained from the practice and publicly available QOF records, so that the results can be interpreted in the correct context. These results will

For peer review only

also be compared to national records of disease profiling using the QOF disease registers and associated prevalence.

Data analysis

Data will be analysed using a fixed effects model with interaction terms. The interaction term will be whether a drug pair is appropriate or inappropriate as defined using the BNF, the current gold standard method[67], with a variable of presence or absence of condition. Pairwise combinations of all conditions will be explored, as the literature suggests that each combination of two diseases has an evidence base. Our tool will complement existing prescribing support tools [68,69] as it places prescribing support within the context of navigating multiple care pathways.

Study outcomes

The primary outcome for phase two is:

- To elicit the proportion of patients with pairwise condition combinations who have been prescribed two or more drugs concurrently, and the type of interaction, if any, between the two.

Further secondary outcomes for this work are to elicit:

- The total number, and severity, of interactions for each clinical condition as defined by the latest gold standard references.
- To compare the numbers of patients identified with these clinical conditions with the practice disease register and to the national reported prevalence using the latest available QOF data.

For peer review only

DISCUSSION

This study protocol is using a mixed methods approach to better understand the challenges associated with multimorbidity and polypharmacy. In determining the utilisation of guidelines in the management of multiple morbidities we will use two methodological approaches. Both Theoretical Domains Framework[29,31–36,62,63] and Normalisation Process Theory have been rigorously evaluated and used within the National Health Service (NHS) context.[38,64–66] The dual analysis will enable a more robust understanding of the current issues. By extracting prescribing data on multimorbid patients for the first time we will be able to quantify the prevalence of polypharmacy in patients with combinations of some of the most common conditions. We will use this work to improve the implementability and acceptability of the new computer tool that is being formed as part of the MITCON grant. The MITCON tool will automatically detect conflict between guidelines to give healthcare professionals the most accurate and up to date information at the point of patient contact.

There are also a number of potential limitations with our approach. Firstly, there is a risk of responder bias at both the practice recruitment and interviewee recruitment stages. Our findings will not necessarily be representative of the whole of the UK however by conducting our study in the West Midlands we have the opportunity to recruit from a range of practices in terms of size and socio-economic environment and staff with a range of seniority and experience. Also, by conducting semi-structured interviews with individual members of staff we expect to reduce the impact of social desirability bias. Furthermore, the majority of our interviews in primary care are running the clinical system EMIS which may have different challenges to the other clinical systems currently running. We seek to

For peer review only

address this by recruiting at a specialist educational meeting to include at least two GPs running a different clinical system. Also, during the training period, it is likely that GPs will have used other systems, so we will prompt around this area so that this limitation is minimised. Also, as this is a pilot study and we are not yet able to accurately estimate numbers of interactions we will focus upon pairwise comparison initially. However, if it becomes apparent that many patients are taking three or four drugs at the same time that interact, we will analyse these separately. Finally, we are aware of larger studies assessing polypharmacy[70,71] but to the best of our knowledge we are the first study to undertake a mixed methods approach within each primary care site.

The findings of each phase will be important in their own right but taken together they will provide a rigorous and robust description of the current issues around managing multimorbidity and the prevalence of polypharmacy. This information will prove invaluable in the development of our novel software tool to ensure that it can be tailored to barriers most amenable to change.

ETHICS AND DISSEMINATION

Ethics

Favourable ethical approval has been granted by The University of Birmingham Research Ethics Committee (ERN_16-0074) on 17th May 2016.

Dissemination

For peer review only

Our work will be disseminated through peer reviewed literature, trade journals, and conferences. We will also use the dedicated web page hosted by the University to serve as a central point of contact and as a repository of our findings. We aim to produce a minimum of three articles from this work to contribute to the international scientific literature.

DECLARATIONS

Trial status

At the time of submission, this study was actively recruiting centres and participants and no analysis work has yet been undertaken.

Authors' contributions

RB wrote the first draft of the protocol and all authors participated in further drafting. AMT provided specialist clinical knowledge. PW and ML provided specialist IT knowledge for the analysis of the polypharmacy data. RB and IL conceived the original idea for this study. All authors read and approved the final manuscript.

Competing interests

None declared.

Funding

This work was supported by the Engineering and Physical Sciences Research Council (Grant No. EP/M014401/1).

Data sharing

For peer review only

Not applicable.

Acknowledgements

This work forms part of the grant 'Automated Conflict Resolution in Clinical Pathways' (EP/M014401/1) which has been funded by the Engineering and Physical Sciences Research Council (EPSRC). The custom built code was funded by The University of Birmingham College of Medical and Dental Science Research Development Fund.

Abbreviations

Chronic kidney Disease (CKD); Chronic Obstructive Pulmonary Disease (COPD); Coronary Heart Disease (CHD); General Practioner (GP); Good Clinical Practice (GCP); National Health Service (NHS); National Institute of Clinical Excellence (NICE); Normalisation Process Theory (NPT); Non-Steroidal Anti-inflammatory Drugs (NSAIDS); Proton Pump Inhibitors (PPIs); Quality Outcomes Framework (QOF); Theoretical Domains Framework (TDF); United Kingdom (UK).

Figure 1 legend: Summary of participation from each of the NHS sectors with analysis overview from the quantitative and qualitative methodology.

REFERENCES

1 Eccles MP, Grimshaw JM, Shekelle P, *et al.* Developing clinical practice guidelines: target audiences, identifying topics for guidelines, guideline group composition and functioning and conflicts of interest. *Implement Sci* 2012;**7**:60. doi:10.1186/1748-5908-7-60

For peer review only

2 Shekelle P, Woolf S, Grimshaw JM, *et al.* Developing clinical practice guidelines: reviewing, reporting, and publishing guidelines; updating guidelines; and the emerging issues of enhancing guideline implementability and accounting for comorbid conditions in guideline development. *Implement Sci* 2012;**7**:62. doi:10.1186/1748-5908-7-62

3 Woolf S, Schünemann HJ, Eccles MP, *et al.* Developing clinical practice guidelines: types of evidence and outcomes; values and economics, synthesis, grading, and presentation and deriving recommendations. *Implement Sci* 2012;**7**:61. doi:10.1186/1748-5908-7-61

4 Campbell H, Hotchkiss R, Bradshaw N, *et al.* Integrated care pathways. *BMJ* 1998;**316**:133–7. doi:10.1136/bmj.316.7125.133

5 Hawley ST, Zikmund-Fisher B, Ubel P, *et al.* The impact of the format of graphical presentation on health-related knowledge and treatment choices. *Patient Educ Couns* 2008;**73**:448–55. doi:10.1016/j.pec.2008.07.023

6 Negrini S, Giovannoni S, Minozzi S, *et al*. Diagnostic therapeutic flow-charts for low back pain patients: the Italian clinical guidelines. *Europa Medicophysica* 2006 June;**42**(2):151-70. http://www.minervamedica.it/en/journals/europa-medicophysica/article.php?cod=R33Y2006N02A0151 (accessed May 2017).

7 Li W, Liu K, Yang H, *et al.* Integrated clinical pathway management for medical quality improvement – based on a semiotically inspired systems architecture. *Eur J Inf Syst* 2014;**23**:400–17. doi:10.1057/ejis.2013.9

8 Schedlbauer A, Prasad V, Mulvaney C, *et al.* What evidence supports the use of computerized alerts and prompts to improve clinicians' prescribing behavior? *J Am Med Inform Assoc* 2009;**16**:531–8. doi:10.1197/jamia.M2910

For peer review only

9 Roshanov PS, Misra S, Gerstein HC, *et al.* Computerized clinical decision support systems for chronic disease management: A decision-maker-researcher partnership systematic review. *Implement Sci* 2011;**6**:92. doi:10.1186/1748-5908-6-92

10 Roshanov PS, Fernandes N, Wilczynski JM, *et al.* Features of effective computerised clinical decision support systems: meta-regression of 162 randomised trials. *BMJ* 2013;**346**:f657. doi:10.1136/bmj.f657

11 Kesselheim AS, Cresswell K, Phansalkar S, *et al.* Clinical decision support systems could be modified to reduce 'alert fatigue' while still minimizing the risk of litigation. *Health Aff (Millwood)* 2011;**30**:2310–7. doi:10.1377/hlthaff.2010.1111

12 National Institute for Health and Care Excellence. Guidance and advice list. http://www.nice.org.uk/guidance/published?type=guidelines (accessed May 2017).

13 Smith SM, Soubhi H, Fortin M, *et al.* Managing patients with multimorbidity: systematic review of interventions in primary care and community settings. *BMJ* 2012;**345**:e5205–e5205. doi:10.1136/bmj.e5205

14 Department of Health. Long Term Conditions Compendium of Information: Third Edition. UK Government. 2012. https://www.gov.uk/government/publications/long-term-conditions-compendium-of-information-third-edition (accessed May 2017).

15 Dumbreck S, Flynn A, Nairn M, *et al.* Drug-disease and drug-drug interactions: systematic examination of recommendations in 12 UK national clinical guidelines. *BMJ* 2015;**350**:h949. doi:10.1136/bmj.h949

16 Hughes LD, McMurdo MET, Guthrie B. Guidelines for people not for diseases: the challenges of applying UK clinical guidelines to people with multimorbidity. *Age Ageing* 2013;**42**:62–9. doi:10.1093/ageing/afs100

17 Guthrie B, Payne K, Alderson P, *et al.* Adapting clinical guidelines to take account of

For peer review only

multimorbidity. *BMJ* 2012;**345**:e6341. doi:10.1136/bmj.e6341

18 Fried TR, Tinetti ME, Iannone L. Primary Care Clinicians' Experiences With Treatment Decision Making for Older Persons With Multiple Conditions. *Arch Intern Med* 2010;**171**:75–80. doi:10.1001/archinternmed.2010.318

19 Martin Duerden, Tony Avery, Rupert Payne. Polypharmacy and medicines optimisation. *The King's Fund* 2013. http://www.volgmed.ru/uploads/files/2014-12/35099-prilozhenie_1-3_k_informacionnomu_pismu_2.pdf (accessed May 2017).

20 Guthrie B, McCowan C, Davey P, *et al.* High risk prescribing in primary care patients particularly vulnerable to adverse drug events: cross sectional population database analysis in Scottish general practice. *BMJ* 2011;**342**:d3514. doi:10.1136/bmj.d3514

21 Sinnott C, Bradley C, Bradley CP. Multimorbidity or polypharmacy: two sides of the same coin? *J Comorbidity* 2015;**5**:29–31. doi:10.15256/joc.2015.5.51

22 Sinnott C, Hugh SM, Boyce MB, *et al.* What to give the patient who has everything? A qualitative study of prescribing for multimorbidity in primary care. *Br J Gen Pract* 2015;**65**:e184–91. doi:10.3399/bjgp15X684001

23 Payne RA, Abel GA, Avery AJ, *et al.* Is polypharmacy always hazardous? A retrospective cohort analysis using linked electronic health records from primary and secondary care. *Br J Clin Pharmacol* 2014;**77**:1073–82. doi:10.1111/bcp.12292

24 Cahir C, Bennett K, Teljeur C, *et al.* Potentially inappropriate prescribing and adverse health outcomes in community dwelling older patients. *Br J Clin Pharmacol* 2014;**77**:201–10. doi:10.1111/bcp.12161

25 Bemben NM. Deprescribing: An Application to Medication Management in Older Adults. *Pharmacother J Hum Pharmacol Drug Ther* 2016;**36**:774–80. doi:10.1002/phar.1776

For peer review only

26 Twigg MJ, Wright D, Barton GR, *et al.* The four or more medicines (FOMM) support service: results from an evaluation of a new community pharmacy service aimed at over-65s. *Int J Pharm Pract* 2015;**23**:407–14. doi:10.1111/ijpp.12196

27 Sinnige J, Braspenning JC, Schellevis FG, *et al.* Inter-practice variation in polypharmacy prevalence amongst older patients in primary care. *Pharmacoepidemiol Drug Saf* 2016;**25**:1033–41. doi:10.1002/pds.4016

28 Kenning C, Fisher L, Bee P, *et al.* Primary care practitioner and patient understanding of the concepts of multimorbidity and self-management: A qualitative study. *SAGE open Med* 2013;**1**:2050312113510001. doi:10.1177/2050312113510001

29 Cane J, O'Connor D, Michie S. Validation of the theoretical domains framework for use in behaviour change and implementation research. *Implement Sci* 2012;**7**:37. doi:10.1186/1748-5908-7-37

30 Michie S, Johnston M, Francis J, *et al.* From Theory to Intervention: Mapping Theoretically Derived Behavioural Determinants to Behaviour Change Techniques. *Appl Psychol* 2008;**57**:660–80. doi:10.1111/j.1464-0597.2008.00341.x

31 Patey AM, Islam R, Francis JJ, *et al.* Anesthesiologists' and surgeons' perceptions about routine pre-operative testing in low-risk patients: application of the Theoretical Domains Framework (TDF) to identify factors that influence physicians' decisions to order pre-operative tests. *Implement Sci* 2012;**7**:52. doi:10.1186/1748-5908-7-52

32 Curran JA, Brehaut J, Patey AM, *et al.* Understanding the Canadian adult CT head rule trial: use of the theoretical domains framework for process evaluation. *Implement Sci* 2013;**8**:25. doi:10.1186/1748-5908-8-25

33 Duncan EM, Francis JJ, Johnston M, *et al.* Learning curves, taking instructions, and patient safety: using a theoretical domains framework in an interview study to

investigate prescribing errors among trainee doctors. *Implement Sci* 2012;**7**:86. doi:10.1186/1748-5908-7-86

34 French SD, Green SE, O'Connor DA, *et al.* Developing theory-informed behaviour change interventions to implement evidence into practice: a systematic approach using the Theoretical Domains Framework. *Implement Sci* 2012;**7**:38. doi:10.1186/1748-5908-7-38

35 Islam R, Tinmouth AT, Francis JJ, *et al.* A cross-country comparison of intensive care physicians' beliefs about their transfusion behaviour: a qualitative study using the Theoretical Domains Framework. *Implement Sci* 2012;**7**:93. doi:10.1186/1748-5908-7-93

36 Bussières AE, Patey AM, Francis JJ, *et al.* Identifying factors likely to influence compliance with diagnostic imaging guideline recommendations for spine disorders among chiropractors in North America: a focus group study using the Theoretical Domains Framework. *Implement Sci* 2012;**7**:82. doi:10.1186/1748-5908-7-82

37 Michie S, Richardson M, Johnston M, *et al.* The behavior change technique taxonomy (v1) of 93 hierarchically clustered techniques: building an international consensus for the reporting of behavior change interventions. *Ann Behav Med* 2013;**46**:81–95. doi:10.1007/s12160-013-9486-6

38 May C, Finch T. Implementing, Embedding, and Integrating Practices: An Outline of Normalization Process Theory. *Sociology* 2009;**43**:535–54. doi:10.1177/0038038509103208

39 Stage KB, Middelboe T, Stage TB, *et al.* Depression in COPD – management and quality of life considerations. *Int J Chron Obstruct Pulmon Dis* 2006;**1**:315-20. http://www.pubmedcentral.nih.gov/articlerender.fcgi?artid=2707161&tool=pmcentr

For peer review only

ez&rendertype=abstract (accessed May 2017).

40 Rana JS, Mittleman MA, Sheikh J, *et al.* Chronic Obstructive Pulmonary Disease, Asthma, and Risk of Type 2 Diabetes in Women. *Diabetes Care* 2004;**27**:2478–84. doi:10.2337/diacare.27.10.2478

41 Lee TA, Pickard AS, Bartle B, *et al.* Osteoarthritis: a comorbid marker for longer life? *Ann Epidemiol* 2007;**17**:380–4. doi:10.1016/j.annepidem.2007.01.033

42 Chaouat A, Naeije R, Weitzenblum E. Pulmonary hypertension in COPD. *Eur Respir J* 2008;**32**:1371–85. doi:10.1183/09031936.00015608

43 Campo G, Pavasini R, Malagù M, *et al.* Chronic obstructive pulmonary disease and ischemic heart disease comorbidity: overview of mechanisms and clinical management. *Cardiovasc Drugs Ther* 2015;**29**:147–57. doi:10.1007/s10557-014-6569-y

44 Mezuk B, Eaton WW, Albrecht S, *et al.* Depression and type 2 diabetes over the lifespan: a meta-analysis. *Diabetes Care* 2008;**31**:2383–90. doi:10.2337/dc08-0985

45 Lin EHB. Depression and osteoarthritis. *Am J Med* 2008;**121**:S16-9. doi:10.1016/j.amjmed.2008.09.009

46 Rubio-Guerra AF, Rodriguez-Lopez L, Vargas-Ayala G, *et al.* Depression increases the risk for uncontrolled hypertension. *Exp Clin Cardiol* 2013;**18**:10–2. http://www.pubmedcentral.nih.gov/articlerender.fcgi?artid=3716493&tool=pmcentrez&rendertype=abstract (accessed May 2017).

47 Rivelli S, Jiang W. Depression and ischemic heart disease: what have we learned from clinical trials? *Curr Opin Cardiol* 2007;**22**:286–91. doi:10.1097/HCO.0b013e3281ead011

48 Berenbaum F. Diabetes-induced osteoarthritis: from a new paradigm to a new

For peer review only

phenotype. *Postgrad Med J* 2012;**88**:240–2. doi:10.1136/pgmj.2010.146399rep

49 Cheung BMY, Li C. Diabetes and hypertension: is there a common metabolic pathway? *Curr Atheroscler Rep* 2012;**14**:160–6. doi:10.1007/s11883-012-0227-2

50 Juutilainen A, Lehto S, Ronnemaa T, *et al.* Type 2 Diabetes as a 'Coronary Heart Disease Equivalent': An 18-year prospective population-based study in Finnish subjects. *Diabetes Care* 2005;**28**:2901–7. doi:10.2337/diacare.28.12.2901

51 Mackenzie IS, MacDonald TM. Treatment of osteoarthritis in hypertensive patients. *Expert Opin Pharmacother* 2010;**11**:393–403. doi:10.1517/14656560903496422

52 Rahman MM, Kopec JA, Cibere J, *et al.* The relationship between osteoarthritis and cardiovascular disease in a population health survey: a cross-sectional study. *BMJ Open* 2013;**3**. doi:10.1136/bmjopen-2013-002624

53 Špinar J. Hypertension and ischemic heart disease. *Cor Vasa* 2012;**54**:e433–8. doi:10.1016/j.crvasa.2012.11.002

54 Palinkas LA, Horwitz SM, Green CA, *et al.* Purposeful Sampling for Qualitative Data Collection and Analysis in Mixed Method Implementation Research. *Adm Policy Ment Health* 2015;**42**:533–44. doi:10.1007/s10488-013-0528-y

55 Paulhus DL. Measurement and Control of Response Bias. In: Robinson J, editor. *Measures of Personality and Social Psychological Attitudes*. Elsevier; 1991. p. 17–59. doi:10.1016/B978-0-12-590241-0.50006-X

56 Berg S. Snowball Sampling—I. In: Kotz S, editor. *Encyclopedia of Statistical Sciences*. John Wiley & Sons, Inc.; 2004. doi:10.1002/0471667196.ess2478.pub2

57 Mason M. Sample Size and Saturation in PhD Studies Using Qualitative Interviews. *Forum Qual Sozialforsch / Forum Qual Soc Res* 2010;**11**.

58 Glaser BG. The Constant Comparative Method of Qualitative Analysis. *Social Problems*

For peer review only

1965;**12**:436-45.

59 Green J, Thorogood N. *Qualitative Methods for Health Research*. SAGE Publications; 2013. https://books.google.com/books?hl=en&lr=&id=qg0tAgAAQBAJ&pgis=1 (accessed May 2017).

60 *Qualitative Researching with Text, Image and Sound: A Practical Handbook for Social Research*. SAGE Publications; 2000. https://books.google.com/books?hl=en&lr=&id=Ab9Z1Js2LDoC&pgis=1 (accessed May 2017.

61 Romney AK, Weller SC, Batchelder WH. Culture as Consensus: A Theory of Culture and Informant Accuracy. *Source Am Anthropol New Ser* 1986;**88**:313-38.http://www.jstor.org/stable/677564 (accessed May 2017).

62 Tavender EJ, Bosch M, Gruen RL, *et al.* Understanding practice: the factors that influence management of mild traumatic brain injury in the emergency department--a qualitative study using the Theoretical Domains Framework. *Implement Sci* 2014;**9**:8. doi:10.1186/1748-5908-9-8

63 Boscart VM, Fernie GR, Lee JH, *et al.* Using psychological theory to inform methods to optimize the implementation of a hand hygiene intervention. *Implement Sci* 2012;**7**:77. doi:10.1186/1748-5908-7-77

64 Finch T. Teledermatology for chronic disease management: coherence and normalization. *Chronic Illn* 2008;**4**:127–34. doi:10.1177/1742395308092483

65 May C. Towards a general theory of implementation. *Implement Sci* 2013;**8**:18. doi:10.1186/1748-5908-8-18

66 May C, Rapley T, Mair FS, *et al*. Normalization Process Theory On-line Users' Manual, Toolkit and NoMAD instrument. http://www.normalizationprocess.org/bibliography/

For peer review only

(accessed May 2017).

67 BNF Publications. https://www.bnf.org/ (accessed May 2017).

68 O'Mahony D, O'Sullivan D, Byrne S, *et al.* STOPP/START criteria for potentially inappropriate prescribing in older people: version 2. *Age Ageing* 2015;**44**:213–8. doi:10.1093/ageing/afu145

69 ScriptSwitch. Prescribing Decision Support for the NHS by ScriptSwitch. http://www.scriptswitch.com/ (accessed May 2017).

70 Guthrie B, Makubate B, Hernandez-Santiago V, *et al.* The rising tide of polypharmacy and drug-drug interactions: population database analysis 1995–2010. *BMC Med* 2015;**13**:74. doi:10.1186/s12916-015-0322-7

71 Moriarty F, Hardy C, Bennett K, *et al.* Trends and interaction of polypharmacy and potentially inappropriate prescribing in primary care over 15 years in Ireland: a repeated cross-sectional study. *BMJ Open* 2015;**5**:e008656. doi:10.1136/bmjopen-2015-008656

For peer review only

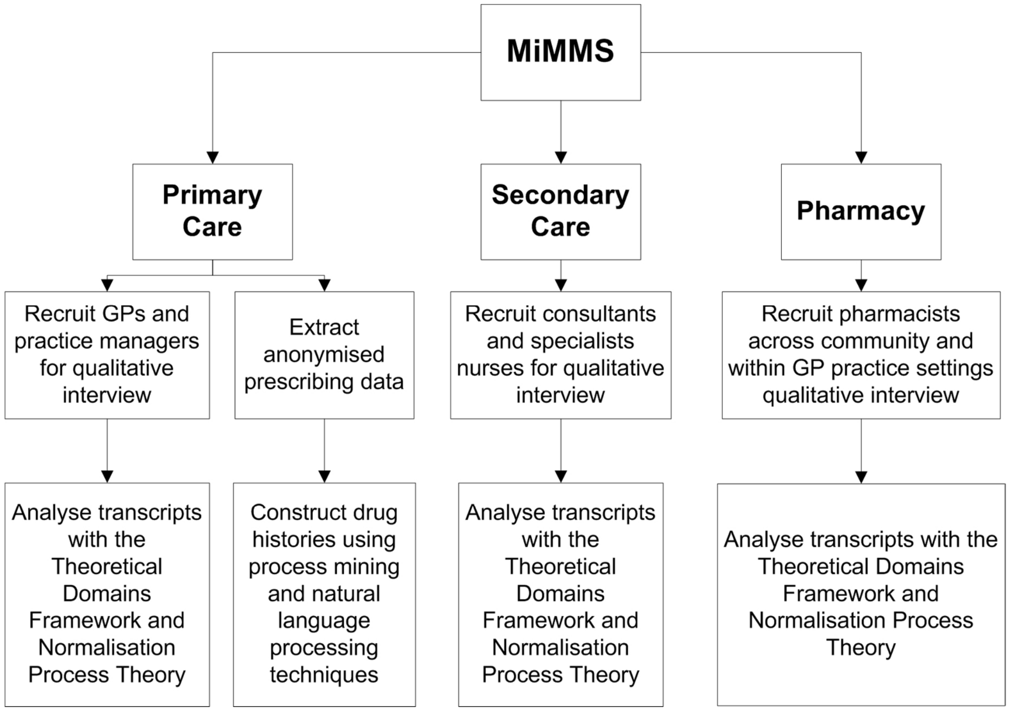

Figure 1: Flow diagram of MiMMS

156x110mm (300 x 300 DPI)

Supplementary file 1 Read codes

Condition	Included (including exception reporting)	Excluded
Hypertension	G2...<br>G20..%<br>G24.. - G2z.. (Excluding G24z1, G2400, G2410, G27..)<br>Gyu2.<br>Gyu20	21261<br>212K.
COPD	H3...<br>H31..% (excluding H3101, H31y0, H3122)<br>H32..%<br>H36.. - H3z.. (excluding H3y0., H3y1.)<br>H4640<br>H4641<br>H5832<br>Hyu30<br>Hyu31	2126F
Depression	E0013,<br>E0021<br>E112.%,<br>E113.%<br>E118.,<br>E11y2<br>E11z2,<br>E130.<br>E135.<br>E2003<br>E291.,<br>E2B..<br>E2B1.,<br>Eu204<br>Eu251<br>Eu32.% (excluding Eu32A, Eu32B, Eu329) Eu33.%<br>Eu341<br>Eu412	212S.
Diabetes	C10..,<br>C109J,<br>C109K,<br>C10C.,<br>C10D.,<br>C10E.%,<br>C10F.% (Excluding C10F8),	21263<br>212H.

For peer review only

	C10G.%,<br>C10H.%,<br>C10M.%,<br>C10N.%,<br>PKyP.,<br>C10P.%,<br>C10Q.	
Hypertension	G2...<br>G20..%<br>G24.. - G2z.. (Excluding G24z1, G2400, G2410, G27..)<br>Gyu2.<br>Gyu20	21261<br>212K.
Osteoarthritis	N05..00 Osteoarthritis and allied disorders	
CKD	1Z12.<br>1Z13.<br>1Z14.<br>1Z15.<br>1Z16.<br>1Z1B. - 1Z1L.<br>1Z1T.<br>1Z1V.<br>1Z1W.<br>1Z1X.<br>1Z1Y.<br>1Z1Z.<br>1Z1a.<br>1Z1b.<br>1Z1c.<br>1Z1d.<br>1Z1e.<br>1Z1f.<br>K053.<br>K054.<br>K055.<br>1Z10.<br>1Z11.<br>1Z17. - 1Z1A.<br>1Z1M.<br>1Z1Q.<br>K051.<br>K052.<br>1Z1N.<br>1Z1P.<br>1Z1R.<br>1Z1S.	2126E

For peer review only

BMJ Open

Assessing the extent of drug interactions amongst patients with multimorbidity in primary and secondary care in the West Midlands (UK): A study protocol for the Mixed Methods Multimorbidity Study (MiMMS)

Journal:	*BMJ Open*
Manuscript ID	bmjopen-2017-016713.R2
Article Type:	Protocol
Date Submitted by the Author:	26-Jun-2017
Complete List of Authors:	Backman, Ruth; University of Birmingham, Institute of Applied Health Research<br>Weber, Philip; University of Birmingham, School of Computer Science<br>Turner, Alice; University of Birmingham, Institute of Inflammation & Aging; Heart of England NHS Foundation Trust<br>Lee, Mark; University of Birmingham, School of Computer Science<br>Litchfield, Ian; University of Birmingham, Institute of Applied Health Research
**Primary Subject Heading**:	Medical management
Secondary Subject Heading:	Evidence based practice, Qualitative research, Pharmacology and therapeutics
Keywords:	Protocol, Multimorbidity, Polypharmacy, NHS, PRIMARY CARE, SECONDARY CARE

SCHOLARONE™
Manuscripts

For peer review only

Assessing the extent of drug interactions amongst patients with multimorbidity in primary and secondary care in the West Midlands (UK): A study protocol for the Mixed Methods Multimorbidity Study (MiMMS)

Ruth Backman[1], Philip Weber[2], Alice M Turner[3,4], Mark Lee[2], Ian Litchfield[1‡].

[1] Institute of Applied Health Research, University of Birmingham, Edgbaston, Birmingham B15 2TT

[2] School of Computer Science, University of Birmingham, Edgbaston, Birmingham B15 2TT

[3] Heartlands Hospital, Heart of England NHS Foundation Trust, Bordesley Green East, Birmingham B9 5SS

[4] Institute of Inflammation & Aging, University of Birmingham, Edgbaston, Birmingham B15 2WB

[‡] Corresponding author

Address: Institute of Applied Health Research, College of Medical and Dental Sciences
University of Birmingham, Edgbaston, Birmingham B15 2TT
United Kingdom

E-mail: i.litchfield@bham.ac.uk

Tel: +44 (0)121 414 6006

Word count: 3585

Word count (Abstract): 288

For peer review only

ABSTRACT

Introduction: The numbers of patients with three or more chronic conditions (multimorbidity) are increasing, and will rise to 2.9 million by 2018 in the United Kingdom alone. Currently in the UK, conditions are mainly managed using over 250 sets of single condition guidance, which has the potential to generate conflicting recommendations for lifestyle and concurrent medication for individual patients with more than one condition. To address some of these issues, we are developing a new computer-based tool to help manage these patients more effectively. For this tool to be applicable and relevant to current practice we must first better understand how existing patients with multimorbidity are being managed, particularly relating to concerns over prescribing and potential polypharmacy.

Methods and analysis: Up to four secondary care centres, two community pharmacies and between four to eight primary care centres in the West Midlands will be recruited. Interviewees will be purposively sampled from these sites, up to a maximum of 30. In this mixed methods study (MiMMS), we will perform a dual framework analysis on the qualitative data; the first analysis will use the Theoretical Domains Framework to assess barriers and enablers for healthcare professionals around the management of multimorbid patients; the second analysis will use Normalisation Process Theory to understand how interventions are currently being successfully implemented in both settings. We will also extract quantitative anonymised patient data from primary care to determine the extent of polypharmacy currently present for patients with multimorbidity in the West Midlands.

For peer review only

Discussion: We aim to combine these data so that we can build a useful, fully implementable tool which addresses the barriers most amenable to change within both primary and secondary care contexts.

Protocol registration: NIHR Clinical Research Network Portfolio Registration CPMS ID 30613.

Strengths and limitations of this study:

- We are using a series of semi-structured interviews to elicit current barriers found within both primary and secondary care when managing patients with multimorbidity.
- This will be complement by the first quantitative exploration of polypharmacy in multimorbid patients in primary care by interrogating routinely collected prescribing data within the same practices.
- This pilot study is based in the West Midlands whose diverse population mean that a range of practices and clinicians will be participating.

Keywords: Protocol; Multimorbidity; Polypharmacy; NHS; Primary care; Secondary care.

INTRODUCTION

In the United Kingdom (UK) there is a focus on the formation and implementation of guidelines to help standardise and improve the quality of care.[1–3] Providers may choose to translate this guidance into local policy to help implementation, for example, a

For peer review only

multidisciplinary care plan which details essential steps in the management of patients with a specific clinical problem.[4] These pathways frequently use graphical descriptions of evidence and options and are typically represented in a single, or series of, flow charts[5,6] and have been shown to have the possibility to improve care if they are implemented correctly.[7] These systems will contain prompts or reminders which in isolation can help to improve practice[8–10] but in combination can lead to alert fatigue.[11]

Of the 250 clinical guidelines published by the National Institute for Clinical Excellence (NICE),[12] only four account for more than one condition. This presents issues for clinicians attempting to use this guidance to manage multimorbidity. Defined as two or more chronic conditions in the same individual,[13] patients with multimorbidity are becoming more prevalent and it is estimated that by 2018 the number of people in the UK with three or more long term conditions will have grown to 2.9 million.[14] Using multiple single conditions guidelines means patients can be left with a significant treatment burden[15,16] and conflicting advice around lifestyle factors.[17] There are also implications for clinicians who struggle to balance the requirements of several guidelines for multimorbid patients, hindered by the lack of outcome data.[18]

One significant consequence of following multiple guidelines is the concurrent prescribing of multiple medications. This phenomenon has been termed “polypharmacy”[19] and has been identified as a complicating issue for patients with multimorbidity.[20] The chances of polypharmacy greatly increase when several sets of guidance are being implemented in patients with multimorbidity[21] and healthcare professionals recognise that fulfilling multiple guidelines might compromise patient centred care.[22] There is evidence this

For peer review only

simultaneous medication administration can lead to an increased risk of patient hospitalisation[23,24] and work has begun to reduce this risk,[25] focussing on reducing inappropriate prescribing of non-steroidal anti-inflammatory drugs (NSAIDS), proton pump inhibitors (PPIs), and duplicate therapy.[26] However progress is inconsistent and large inter-practice variation in levels of polypharmacy remains.[27]

In response to the issues raised in managing increasing numbers of multimorbid patients there have been calls in the UK for improved integration of existing guidelines[28] with the maxim 'treat the patient, not the disease'. In response the MITCON project has begun which involves the development of a new computer based tool which will automatically detect conflict between guidelines.[Litchfield I, Bordbar B, Bowles J, et al. A protocol for the development of automated conflict resolution between multiple clinical pathways. 2017. Unpublished] To ensure that this tool is applicable and relevant we need to understand how multiple guidelines are being translated into existing systems and processes and the extent and implications for patients and care providers of prescribing multiple medications. This protocol details the **Mi**xed **M**ethods **M**ultimorbidity **S**tudy (MiMMS) which seeks to both identify barriers and facilitators to successful guideline implementation, and determine current levels of polypharmacy in multimorbid patients in the West Midlands.

Research Questions

There are two distinct yet related research questions that we will answer. The first concerns the way in which guidelines are used in managing multimorbid patients in two settings – primary and secondary care. The second will involve a quantitative assessment of polypharmacy in multimorbid patients in primary care. Although previous work has

For peer review only

explored multimorbidity, ours is the first to look at both primary and secondary care settings and corroborate qualitative work with quantitative data on polypharmacy at the same GP practices.

1. Managing multimorbidity

We will use the Theoretical Domains Framework (TDF)[29,30] to identify barriers around managing patients with multimorbidity. This framework has been extensively validated within this setting,[31–36] and allows linkage of behaviour change techniques[30,37] if required later in the project. We will also use Normalisation Process Theory (NPT)[38] to identify the current successful implementation strategies for guidelines and associated computer based tools used within current practice. This theory has also been extensively validated and can assess the process whereby new items become routine practice. To the best of our knowledge, this work is the first to use a dual framework approach for analysis. Furthermore, this work will seek to identify barriers in multiple NHS settings so that the full patient pathway with a number of healthcare professionals can be explored.

2. Prevalence of multimorbidity

We aim to assess current levels of polypharmacy for up to six chronic conditions within primary care retrospectively over a 24 month period. In doing so, we will determine the percentage of patients that experience polypharmacy and whether there are patients with certain combinations of conditions that are most at risk. The literature indicates that our target conditions will occur in at least a pairwise combination. We will therefore perform pairwise comparisons of conditions to assess numbers of patients who have interactions within their prescribed medications. [39–53] This will allow us to determine which

For peer review only

combinations of conditions are most frequently associated with polypharmacy. For this work we are defining multimorbidity as having two or more of our target conditions diagnosed with an active Read code. We are defining polypharmacy as taking a minimum of two drugs at any one time point so that we can assess the interaction.

METHODS AND ANALYSIS

Settings and Participants

Site recruitment

We will assess all primary care centres in the West Midlands for study eligibility (defined as using EMIS as their clinical system). We will also purposively sample up to four secondary care centres with differing patient demographics in the West Midlands. Finally, we will purposively sample up to two pharmacy centres.

Research design

We are using a mixed methods approach in this work and will be conducting the study in two interrelated phases (Figure 1). Phase I is the qualitative exploration of the implementation of current guidelines including the barriers and enablers to the management of multimorbid patients in a primary and secondary care settings including the use of clinical software systems. Phase II will use quantitative methods to explore the correlation between clinical condition combinations and the numbers of interacting medications prescribed in primary care.

Figure 1: Flow diagram of MiMMS

For peer review only

Phase I

Site Recruitment

In accordance with the theory of maximum variation,[54] between 4 and 8 primary care sites and up to 4 secondary care sites will be recruited for this pilot from the West Midlands region. This will also help mitigate the risk of responder bias.[55] Sites will be initiated once all research and development (R&D) approvals have been met, and all necessary documents have been returned to the coordinating centre.

Recruitment for these sites will occur through written expressions of interest via the Primary Care Research Network (PCRN) as well as opportunistic recruitment of General Practioners (GPs) during appropriate educational meetings. We will use purposive sampling for the secondary care and community pharmacy sites and will aim to recruit within different working environments e.g. community pharmacy and pharmacists working within a GP practice, where possible. We will also interview healthcare professionals within different departments in secondary care.

Primary care sites that have EMIS web as their clinical system can participate in both phases of the project. We will aim to recruit healthcare professionals who have used other clinical systems such as TPP (SystmOne) or Vision to identify specific barriers relating to these systems.

Healthcare professionals' recruitment

All health care professionals with experience either of managing patients with multimorbidity, or who have implemented new guidelines locally, will be invited to

For peer review only

participate in Phase I. In primary care we expect to recruit fully qualified, and registrar level GPs; advanced nurse practitioners; and practice managers. Within secondary care we will recruit participants from specialities relevant to the 6 diseases targeted in the quantitative work as well as elderly care physicians if possible. These will include but not be limited to consultants; senior registrars (at least ST4 and above); and advanced nurse practitioners. Additionally, we will aim to recruit a minimum of two community pharmacists. All sampling will be performed using an iterative snowballing approach[56].

In each case we will aim to recruit at least two individuals from each of the staff groups stated above, and we will continue with purposive sampling until data saturation has been achieved[57].

Data management

In depth semi structured interviews will be conducted using a topic guide tailored to both the NPT and TDF (Additional file 1) so that both barriers around management of multimorbid patients and current guideline usage can be elicited. Prompts and examples will be used to ensure that data is captured and we will perform constant comparison analysis[58] during the interview process. In depth semi structured interviews will allow these specific topics to be covered, whilst retaining an element of narrative so that healthcare professionals can share the factors that are important to them. Interviewees will always be given the opportunity to add anything they wish at the start and end of the interview. Field notes will be taken by the researcher, both to allow for continued professional development and also to take note of surroundings and reflection of important findings.

For peer review only

Where possible, participants will be sent a copy of the consent form and information sheet at least 24 hours before the interview so that they have time to consider taking part and also time to reflect upon the challenges of managing these types of patients. Using a snowballing technique,[56] sometimes known as the chain referral technique where an interviewee is asked to suggest a member of their team who may have further insights on a particular topic, this may not always be possible, so if participants require less time to decide to take part they may do so.

Fully informed consent will be taken by a trained researcher who holds a current good clinical practice (GCP) certificate and all associated approvals. We recognise that healthcare professionals may feel uncomfortable sharing cases where they feel that patients may not have been managed to the published guidance, however all participants will be reassured that not all questions have to be answered and the interview can stop at any time. Any data that could identify the individual practice, or patient will be anonymised prior to any form of dissemination. Participants will have up to two weeks after the interview to withdraw their data prior to data analysis.

We will aim to conduct 30 interviews, however, interviews will be stopped when data saturation has been reached and no new major themes are arising within the last five transcripts. [59,60] Due to our interview topics being around a specific aspect of care, combined with interviewees having a similar role and training background we should achieve saturation within our recruitment target.[61] We will aim to include at least two interviews from each group of healthcare professionals (Consultants; Specialist Registrars;

For peer review only

GPs; GP registrars; Practice managers; Advanced nurse practitioners; Community pharmacists). Purposive sampling will be used with snowballing to ensure that the correct mix of healthcare professionals is included.

Data Analysis

All interviews will be recorded using a digital voice recorder and consent for this will be obtained prior to starting. Digital recordings will then be transcribed verbatim prior to analysis.

The returned document will be checked, both for queries and to ensure that anonymity is protected prior to being imported into a suitable analysis program such as NVivo (V11).

TDF will be the main conceptual framework used in the qualitative work. This has been validated in a series of primary and secondary studies [29,31–36,62,63] as a method of understanding behaviour within a clinical setting. NPT will also be used and this framework has been developed to support the implementation and evaluation of complex interventions [38,64,65] and has been used extensively in trials, systematic reviews and qualitative work.[66] This theory will allow us to assess the 'key ingredients' when a guideline or local initiative has been successfully been implemented in practice. This information will help us to include ingredients for successful implementation of our new tool to help more effectively manage patients with multimorbidity. Outside of these two frameworks, we will remain sensitive to new themes and will analyse iteratively.

Study Outcomes

For peer review only

The outcomes for the qualitative work are:

- To assess how guidelines and other local initiatives are being implemented in the management of patients with multimorbidity within primary and secondary care with the outcome of defining ‘key ingredients’ for successful implementation; and
- To understand the barriers and enablers around managing multimorbidity, including prescribing including which factors are taken into consideration and how clinical decisions are made.

Phase II

Site recruitment

Primary care centres who have agreed to take part in phase one and have EMIS as their clinical system will be approached for phase two.

Participant recruitment/data collection

We will use custom built code provided by PRIMIS (The University of Nottingham, 15th Floor Tower Building, University Park, Nottingham. NG7 2RD https://www.nottingham.ac.uk/primis/index.aspx) which generates a report within the GPs’ clinical system. The code will be fully tested prior to use to ensure patient anonymity is protected. All eligible patients over the age of 18 who have been registered at the practice for a minimum of three months will have data extracted relating to prescriptions. For patients to be fully eligible they must also have a Read code within their notes at any time for at least one of the target conditions (Supplementary file 1) and have had at least one prescription issued within the last two years. All data will remain anonymous and we will extract one possible identifier, sex, to allow us to assess the population demographic. To

For peer review only

ensure anonymity, age at the date of extraction will also be collected rather than date of birth. The code will produce three reports; one will detail the practice profile, one will detail the conditions the patient has coded with the Read code included, and the final report will detail the drug history over the preceding 24 months.

Sample size

This study uses a retrospective audit approach to collect data, therefore there is limited scope for increasing the sample size as there is not a traditional recruitment component. Using the Quality Outcomes Framework (QOF) data from 2014-2015, the average prevalence and list size has been calculated in Table 1. Currently, there is no register of patients with osteoarthritis, so these numbers are not available. Table 2 shows the estimated numbers extrapolated from Table 1, modelling the effects of practice number. It is likely that these are minimum estimates as patients may have associated Read codes but have not been included on the disease register. Currently we have no data for the number of prescribed drugs that currently interact for this population. Assuming that 10% of drugs prescribed have an interaction, we can assume that a minimum of 53 and a maximum of 806 parameters can be assessed (Table 2) which will be sufficient for the fixed effects model. Therefore in this pilot study we aim to recruit a minimum of four primary care centres up to a maximum of eight with the overall intention of using this data to form the basis of a sample size calculation in a larger study.

For peer review only

Table 1: Estimates within UK primary care between 2014 - 2015

	List size (total number)	List size (estimated 30-74 years)	CHD (Number on register)	Hypertension (Number on register)	COPD (Number on register)	Diabetes Mellitus 17 years and above (Number on register)	Depression (Number on register)
**Average**	7,304	4,049	237	1,007	133	375	425
**Standard Deviation**	4,429	2,446	167	650	98	233	338
**Inter quartile range (IQR)**	5,870	3,323	215	854	119	301	400

Table 2: Minimum and maximum estimates for selected conditions

	4 GP practices	6 GP practices	8 GP practices
Estimated average list	29,216	43,824	58,432
Estimated average list of 30-74	16,196	24,294	32,392
Estimated number of Coronary heart disease patients	948	1,422	1,896
Estimated number of Hypertension patients	4,028	6,042	8,056
Estimated number of COPD patients	532	798	1,064
Estimated number of Diabetes Mellitus patients	1,500	2,250	3,000
Estimated number of Depression patients	1,700	2,550	3,400

For peer review only

Data management

The QOF business rules version 34 will be used to extract Read codes for five of our six target conditions (Chronic Obstructive Pulmonary Disease (COPD), Type 2 Diabetes, Depression, Coronary Heart Disease (CHD), and Hypertension). The general parent code will be used to extract patients suffering from Osteoarthritis as there are currently no QOF indicators that are suitable for this (supplementary file 1). Every patient identified using these codes will have retrospective prescription data collected for the preceding two years. Unique subject identification numbers will be generated so that a comprehensive picture of prescriptions can be built up for each patient, whilst remaining anonymised. The clinical extraction code containing these details will be generated by a company with expertise in this area of work so that only relevant data is extracted and patient anonymity is preserved. We will not use a condition clustering approach and will only use the combinations of conditions as a way to characterise our population rather than drawing conclusions about prevalence of comorbidities.

After the patient's drug history has been constructed using a process mining approach, we will use our new drug interaction tool to assess the numbers and types of conflicts. This tool is based upon the BNF interactions list, so we will use the same categories of no interaction, mild interaction and severe interaction. We will also use the expertise of ML to enable to use of Natural Language Processing within this data set to pull out the types of interaction, and will use any relating to drug clearance to reinterpret those patients with chronic kidney disease. This work will help validate the first module within the larger software based that is being developed within the grant. We will also extract data relating to chronic kidney disease (CKD) stage and we will use this data to interpret our findings due to changes in

For peer review only

renal function often triggering a change in prescriptions. We recognise that there are challenges managing patients with multimorbidity, and that there may be a time when drugs that interact have to be used due to the clinical severity of one or more conditions. Therefore this data is not going to be linked to specific prescribers, and data will be fed back to practices in a pseudo-anonymised form.

A practice age demographic profile will be gained from the practice and publicly available QOF records, so that the results can be interpreted in the correct context. These results will also be compared to national records of disease profiling using the QOF disease registers and associated prevalence.

Data analysis

Data will be analysed using a fixed effects model with interaction terms. The interaction term will be whether a drug pair is appropriate or inappropriate as defined using the BNF, the current gold standard method[67], with a variable of presence or absence of condition. Pairwise combinations of all conditions will be explored, as the literature suggests that each combination of two diseases has an evidence base. Our tool will complement existing prescribing support tools [68,69] as it places prescribing support within the context of navigating multiple care pathways.

Study outcomes

The primary outcome for phase two is:

For peer review only

- To elicit the proportion of patients with pairwise condition combinations who have been prescribed two or more drugs concurrently, and the type of interaction, if any, between the two.

Further secondary outcomes for this work are to elicit:

- The total number, and severity, of interactions for each clinical condition as defined by the latest gold standard references.
- To compare the numbers of patients identified with these clinical conditions with the practice disease register and to the national reported prevalence using the latest available QOF data.

For peer review only

DISCUSSION

This study protocol is using a mixed methods approach to better understand the challenges associated with multimorbidity and polypharmacy. In determining the utilisation of guidelines in the management of multiple morbidities we will use two methodological approaches. Both Theoretical Domains Framework[29,31–36,62,63] and Normalisation Process Theory have been rigorously evaluated and used within the National Health Service (NHS) context. [38,64–66]The dual analysis will enable a more robust understanding of the current issues. By extracting prescribing data on multimorbid patients for the first time we will be able to quantify the prevalence of polypharmacy in patients with combinations of some of the most common conditions. We will use this work to improve the implementability and acceptability of the new computer tool that is being formed as part of the MITCON grant. The MITCON tool will automatically detect conflict between guidelines

to give healthcare professionals the most accurate and up to date information at the point of patient contact.

There are also a number of potential limitations with our approach. Firstly, there is a risk of responder bias at both the practice recruitment and interviewee recruitment stages. Our findings will not necessarily be representative of the whole of the UK however by conducting our study in the West Midlands we have the opportunity to recruit from a range of practices in terms of size and socio-economic environment and staff with a range of seniority and experience. Also, by conducting semi-structured interviews with individual members of staff we expect to reduce the impact of social desirability bias. Furthermore, the majority of our interviews in primary care are running the clinical system EMIS which may have different challenges to the other clinical systems currently running. We seek to address this by recruiting at a specialist educational meeting to include at least two GPs running a different clinical system. Also, during the training period, it is likely that GPs will have used other systems, so we will prompt around this area so that this limitation is minimised. Also, as this is a pilot study and we are not yet able to accurately estimate numbers of interactions we will focus upon pairwise comparison initially. However, if it becomes apparent that many patients are taking three or four drugs at the same time that interact, we will analyse these separately. Furthermore, we are only able to assess six of the common morbidities in the UK, so there is a potential for missing excess morbidity as well as introducing bias relating to our chosen conditions. However, we have chosen prevalent chronic conditions which are all known to interact with each other, both within and between body systems. Finally, we are aware of larger studies assessing polypharmacy

For peer review only

[70,71] but to the best of our knowledge we are the first study to undertake a mixed methods approach within each primary care site.

The findings of each phase will be important in their own right but taken together they will provide a rigorous and robust description of the current issues around managing multimorbidity and the prevalence of polypharmacy. This information will prove invaluable in the development of our novel software tool to ensure that it can be tailored to barriers most amenable to change.

ETHICS AND DISSEMINATION

Ethics

Favourable ethical approval has been granted by The University of Birmingham Research Ethics Committee (ERN_16-0074) on 17th May 2016.

Dissemination

Our work will be disseminated through peer reviewed literature, trade journals, and conferences. We will also use the dedicated web page hosted by the University to serve as a central point of contact and as a repository of our findings. We aim to produce a minimum of three articles from this work to contribute to the international scientific literature.

DECLARATIONS

Trial status

For peer review only

At the time of submission, this study was actively recruiting centres and participants and no analysis work has yet been undertaken.

Authors' contributions

RB wrote the first draft of the protocol and all authors participated in further drafting. AMT provided specialist clinical knowledge. PW and ML provided specialist IT knowledge for the analysis of the polypharmacy data. RB and IL conceived the original idea for this study. All authors read and approved the final manuscript.

Competing interests

None declared.

Funding

This work was supported by the Engineering and Physical Sciences Research Council (Grant No. EP/M014401/1).

Data sharing

Not applicable.

Acknowledgements

This work forms part of the grant 'Automated Conflict Resolution in Clinical Pathways' (EP/M014401/1) which has been funded by the Engineering and Physical Sciences Research Council (EPSRC). The custom built code was funded by The University of Birmingham College of Medical and Dental Science Research Development Fund.

For peer review only

Abbreviations

Chronic kidney Disease (CKD); Chronic Obstructive Pulmonary Disease (COPD); Coronary Heart Disease (CHD); General Practioner (GP); Good Clinical Practice (GCP); National Health Service (NHS); National Institute of Clinical Excellence (NICE); Normalisation Process Theory (NPT); Non-Steroidal Anti-inflammatory Drugs (NSAIDS); Proton Pump Inhibitors (PPIs); Quality Outcomes Framework (QOF); Theoretical Domains Framework (TDF); United Kingdom (UK).

Figure 1 legend: Summary of participation from each of the NHS sectors with analysis overview from the quantitative and qualitative methodology.

REFERENCES

1 Eccles MP, Grimshaw JM, Shekelle P, *et al.* Developing clinical practice guidelines: target audiences, identifying topics for guidelines, guideline group composition and functioning and conflicts of interest. *Implement Sci* 2012;**7**:60. doi:10.1186/1748-5908-7-60

2 Shekelle P, Woolf S, Grimshaw JM, *et al.* Developing clinical practice guidelines: reviewing, reporting, and publishing guidelines; updating guidelines; and the emerging issues of enhancing guideline implementability and accounting for comorbid conditions in guideline development. *Implement Sci* 2012;**7**:62. doi:10.1186/1748-5908-7-62

3 Woolf S, Schünemann HJ, Eccles MP, *et al.* Developing clinical practice guidelines: types of evidence and outcomes; values and economics, synthesis, grading, and

presentation and deriving recommendations. *Implement Sci* 2012;**7**:61. doi:10.1186/1748-5908-7-61

4 Campbell H, Hotchkiss R, Bradshaw N, *et al.* Integrated care pathways. *BMJ* 1998;**316**:133–7. doi:10.1136/bmj.316.7125.133

5 Hawley ST, Zikmund-Fisher B, Ubel P, *et al.* The impact of the format of graphical presentation on health-related knowledge and treatment choices. *Patient Educ Couns* 2008;**73**:448–55. doi:10.1016/j.pec.2008.07.023

6 Diagnostic therapeutic flow-charts for low back pain patients: the Italian clinical guidelines - Europa Medicophysica 2006 June;42(2):151-70 - Minerva Medica - Journals. http://www.minervamedica.it/en/journals/europa-medicophysica/article.php?cod=R33Y2006N02A0151 (accessed 23 Feb2016).

7 Li W, Liu K, Yang H, *et al.* Integrated clinical pathway management for medical quality improvement – based on a semiotically inspired systems architecture. *Eur J Inf Syst* 2014;**23**:400–17. doi:10.1057/ejis.2013.9

8 Schedlbauer A, Prasad V, Mulvaney C, *et al.* What evidence supports the use of computerized alerts and prompts to improve clinicians' prescribing behavior? *J Am Med Inform Assoc* 2009;**16**:531–8. doi:10.1197/jamia.M2910

9 Roshanov PS, Misra S, Gerstein HC, *et al.* Computerized clinical decision support systems for chronic disease management: A decision-maker-researcher partnership systematic review. *Implement Sci* 2011;**6**:92. doi:10.1186/1748-5908-6-92

10 Roshanov PS, Fernandes N, Wilczynski JM, *et al.* Features of effective computerised clinical decision support systems: meta-regression of 162 randomised trials. *BMJ* 2013;**346**:f657. doi:10.1136/bmj.f657

11 Kesselheim AS, Cresswell K, Phansalkar S, *et al.* Clinical decision support systems

For peer review only

could be modified to reduce 'alert fatigue' while still minimizing the risk of litigation. *Health Aff (Millwood)* 2011;**30**:2310–7. doi:10.1377/hlthaff.2010.1111

12 Published | Guidance | NICE. http://www.nice.org.uk/guidance/published?type=guidelines (accessed 23 Feb2016).

13 Smith SM, Soubhi H, Fortin M, *et al.* Managing patients with multimorbidity: systematic review of interventions in primary care and community settings. *BMJ* 2012;**345**:e5205–e5205. doi:10.1136/bmj.e5205

14 Long Term Conditions Compendium of Information: Third Edition - Publications - GOV.UK. https://www.gov.uk/government/publications/long-term-conditions-compendium-of-information-third-edition (accessed 23 Feb2016).

15 Dumbreck S, Flynn A, Nairn M, *et al.* Drug-disease and drug-drug interactions: systematic examination of recommendations in 12 UK national clinical guidelines. *BMJ* 2015;**350**:h949. doi:10.1136/bmj.h949

16 Hughes LD, McMurdo MET, Guthrie B. Guidelines for people not for diseases: the challenges of applying UK clinical guidelines to people with multimorbidity. *Age Ageing* 2013;**42**:62–9. doi:10.1093/ageing/afs100

17 Guthrie B, Payne K, Alderson P, *et al.* Adapting clinical guidelines to take account of multimorbidity. *BMJ* 2012;**345**:e6341. doi:10.1136/bmj.e6341

18 Fried TR, Tinetti ME, Iannone L. Primary Care Clinicians' Experiences With Treatment Decision Making for Older Persons With Multiple Conditions. *Arch Intern Med* 2010;**171**:75–80. doi:10.1001/archinternmed.2010.318

19 Polypharmacy and medicines optimisation. http://www.volgmed.ru/uploads/files/2014-12/35099-prilozhenie_1-3_k_informacionnomu_pismu_2.pdf (accessed 3 Mar2016).

For peer review only

20 Guthrie B, McCowan C, Davey P, *et al.* High risk prescribing in primary care patients particularly vulnerable to adverse drug events: cross sectional population database analysis in Scottish general practice. *BMJ* 2011;**342**:d3514. doi:10.1136/bmj.d3514

21 Sinnott C, Bradley C, Bradley CP. Multimorbidity or polypharmacy: two sides of the same coin? *J Comorbidity* 2015;**5**:29–31. doi:10.15256/joc.2015.5.51

22 Sinnott C, Hugh SM, Boyce MB, *et al.* What to give the patient who has everything? A qualitative study of prescribing for multimorbidity in primary care. *Br J Gen Pract* 2015;**65**:e184–91. doi:10.3399/bjgp15X684001

23 Payne RA, Abel GA, Avery AJ, *et al.* Is polypharmacy always hazardous? A retrospective cohort analysis using linked electronic health records from primary and secondary care. *Br J Clin Pharmacol* 2014;**77**:1073–82. doi:10.1111/bcp.12292

24 Cahir C, Bennett K, Teljeur C, *et al.* Potentially inappropriate prescribing and adverse health outcomes in community dwelling older patients. *Br J Clin Pharmacol* 2014;**77**:201–10. doi:10.1111/bcp.12161

25 Bemben NM. Deprescribing: An Application to Medication Management in Older Adults. *Pharmacother J Hum Pharmacol Drug Ther* 2016;**36**:774–80. doi:10.1002/phar.1776

26 Twigg MJ, Wright D, Barton GR, *et al.* The four or more medicines (FOMM) support service: results from an evaluation of a new community pharmacy service aimed at over-65s. *Int J Pharm Pract* 2015;**23**:407–14. doi:10.1111/ijpp.12196

27 Sinnige J, Braspenning JC, Schellevis FG, *et al.* Inter-practice variation in polypharmacy prevalence amongst older patients in primary care. *Pharmacoepidemiol Drug Saf* 2016;**25**:1033–41. doi:10.1002/pds.4016

28 Kenning C, Fisher L, Bee P, *et al.* Primary care practitioner and patient understanding

For peer review only

of the concepts of multimorbidity and self-management: A qualitative study. *SAGE open Med* 2013;**1**:2050312113510001. doi:10.1177/2050312113510001

29 Cane J, O'Connor D, Michie S. Validation of the theoretical domains framework for use in behaviour change and implementation research. *Implement Sci* 2012;**7**:37. doi:10.1186/1748-5908-7-37

30 Michie S, Johnston M, Francis J, *et al.* From Theory to Intervention: Mapping Theoretically Derived Behavioural Determinants to Behaviour Change Techniques. *Appl Psychol* 2008;**57**:660–80. doi:10.1111/j.1464-0597.2008.00341.x

31 Patey AM, Islam R, Francis JJ, *et al.* Anesthesiologists' and surgeons' perceptions about routine pre-operative testing in low-risk patients: application of the Theoretical Domains Framework (TDF) to identify factors that influence physicians' decisions to order pre-operative tests. *Implement Sci* 2012;**7**:52. doi:10.1186/1748-5908-7-52

32 Curran JA, Brehaut J, Patey AM, *et al.* Understanding the Canadian adult CT head rule trial: use of the theoretical domains framework for process evaluation. *Implement Sci* 2013;**8**:25. doi:10.1186/1748-5908-8-25

33 Duncan EM, Francis JJ, Johnston M, *et al.* Learning curves, taking instructions, and patient safety: using a theoretical domains framework in an interview study to investigate prescribing errors among trainee doctors. *Implement Sci* 2012;**7**:86. doi:10.1186/1748-5908-7-86

34 French SD, Green SE, O'Connor DA, *et al.* Developing theory-informed behaviour change interventions to implement evidence into practice: a systematic approach using the Theoretical Domains Framework. *Implement Sci* 2012;**7**:38. doi:10.1186/1748-5908-7-38

35 Islam R, Tinmouth AT, Francis JJ, *et al.* A cross-country comparison of intensive care

For peer review only

physicians' beliefs about their transfusion behaviour: a qualitative study using the Theoretical Domains Framework. *Implement Sci* 2012;**7**:93. doi:10.1186/1748-5908-7-93

36 Bussières AE, Patey AM, Francis JJ, *et al.* Identifying factors likely to influence compliance with diagnostic imaging guideline recommendations for spine disorders among chiropractors in North America: a focus group study using the Theoretical Domains Framework. *Implement Sci* 2012;**7**:82. doi:10.1186/1748-5908-7-82

37 Michie S, Richardson M, Johnston M, *et al.* The behavior change technique taxonomy (v1) of 93 hierarchically clustered techniques: building an international consensus for the reporting of behavior change interventions. *Ann Behav Med* 2013;**46**:81–95. doi:10.1007/s12160-013-9486-6

38 May C, Finch T. Implementing, Embedding, and Integrating Practices: An Outline of Normalization Process Theory. *Sociology* 2009;**43**:535–54. doi:10.1177/0038038509103208

39 Stage KB, Middelboe T, Stage TB, *et al.* Depression in COPD--management and quality of life considerations. *Int J Chron Obstruct Pulmon Dis* 2006;**1**:315–20.http://www.pubmedcentral.nih.gov/articlerender.fcgi?artid=2707161&tool=pmcentrez&rendertype=abstract (accessed 3 Mar2016).

40 Rana JS, Mittleman MA, Sheikh J, *et al.* Chronic Obstructive Pulmonary Disease, Asthma, and Risk of Type 2 Diabetes in Women. *Diabetes Care* 2004;**27**:2478–84. doi:10.2337/diacare.27.10.2478

41 Lee TA, Pickard AS, Bartle B, *et al.* Osteoarthritis: a comorbid marker for longer life? *Ann Epidemiol* 2007;**17**:380–4. doi:10.1016/j.annepidem.2007.01.033

42 Chaouat A, Naeije R, Weitzenblum E. Pulmonary hypertension in COPD. *Eur Respir J*

For peer review only

2008;**32**:1371–85. doi:10.1183/09031936.00015608

43 Campo G, Pavasini R, Malagù M, *et al.* Chronic obstructive pulmonary disease and ischemic heart disease comorbidity: overview of mechanisms and clinical management. *Cardiovasc Drugs Ther* 2015;**29**:147–57. doi:10.1007/s10557-014-6569-y

44 Mezuk B, Eaton WW, Albrecht S, *et al.* Depression and type 2 diabetes over the lifespan: a meta-analysis. *Diabetes Care* 2008;**31**:2383–90. doi:10.2337/dc08-0985

45 Lin EHB. Depression and osteoarthritis. *Am J Med* 2008;**121**:S16-9. doi:10.1016/j.amjmed.2008.09.009

46 Rubio-Guerra AF, Rodriguez-Lopez L, Vargas-Ayala G, *et al.* Depression increases the risk for uncontrolled hypertension. *Exp Clin Cardiol* 2013;**18**:10–2.http://www.pubmedcentral.nih.gov/articlerender.fcgi?artid=3716493&tool=pmcentrez&rendertype=abstract (accessed 3 Mar2016).

47 Rivelli S, Jiang W. Depression and ischemic heart disease: what have we learned from clinical trials? *Curr Opin Cardiol* 2007;**22**:286–91. doi:10.1097/HCO.0b013e3281ead011

48 Berenbaum F. Diabetes-induced osteoarthritis: from a new paradigm to a new phenotype. *Postgrad Med J* 2012;**88**:240–2. doi:10.1136/pgmj.2010.146399rep

49 Cheung BMY, Li C. Diabetes and hypertension: is there a common metabolic pathway? *Curr Atheroscler Rep* 2012;**14**:160–6. doi:10.1007/s11883-012-0227-2

50 Juutilainen A, Lehto S, Ronnemaa T, *et al.* Type 2 Diabetes as a 'Coronary Heart Disease Equivalent': An 18-year prospective population-based study in Finnish subjects. *Diabetes Care* 2005;**28**:2901–7. doi:10.2337/diacare.28.12.2901

51 Mackenzie IS, MacDonald TM. Treatment of osteoarthritis in hypertensive patients.

For peer review only

Expert Opin Pharmacother 2010;**11**:393–403. doi:10.1517/14656560903496422

52 Rahman MM, Kopec JA, Cibere J, *et al.* The relationship between osteoarthritis and cardiovascular disease in a population health survey: a cross-sectional study. *BMJ Open* 2013;**3**. doi:10.1136/bmjopen-2013-002624

53 Špinar J. Hypertension and ischemic heart disease. *Cor Vasa* 2012;**54**:e433–8. doi:10.1016/j.crvasa.2012.11.002

54 Palinkas LA, Horwitz SM, Green CA, *et al.* Purposeful Sampling for Qualitative Data Collection and Analysis in Mixed Method Implementation Research. *Adm Policy Ment Health* 2015;**42**:533–44. doi:10.1007/s10488-013-0528-y

55 Paulhus DL, L. D. Measurement and Control of Response Bias. In: *Measures of Personality and Social Psychological Attitudes*. Elsevier 1991. 17–59. doi:10.1016/B978-0-12-590241-0.50006-X

56 Berg S. Snowball Sampling—I. In: *Encyclopedia of Statistical Sciences*. John Wiley & Sons, Inc. 2004. doi:10.1002/0471667196.ess2478.pub2

57 Mason M. Sample Size and Saturation in PhD Studies Using Qualitative Interviews. *Forum Qual Sozialforsch / Forum Qual Soc Res* 2010;**11**.

58 The Constant Comparative Method of Qualitative Analysis on JSTOR. http://www.jstor.org/stable/798843?seq=1#page_scan_tab_contents (accessed 25 Feb2016).

59 Green J, Thorogood N. *Qualitative Methods for Health Research*. SAGE Publications 2013. https://books.google.com/books?hl=en&lr=&id=qg0tAgAAQBAJ&pgis=1 (accessed 25 Feb2016).

60 *Qualitative Researching with Text, Image and Sound: A Practical Handbook for Social Research*. SAGE Publications 2000.

For peer review only

https://books.google.com/books?hl=en&lr=&id=Ab9Z1Js2LDoC&pgis=1 (accessed 25 Feb2016).

61 Romney AK, Weller SC, Batchelder WH. Culture as Consensus: A Theory of Culture and Informant Accuracy. *Source Am Anthropol New Ser* 1986;**88**:313–38.http://www.jstor.org/stable/677564 (accessed 22 May2017).

62 Tavender EJ, Bosch M, Gruen RL, *et al.* Understanding practice: the factors that influence management of mild traumatic brain injury in the emergency department--a qualitative study using the Theoretical Domains Framework. *Implement Sci* 2014;**9**:8. doi:10.1186/1748-5908-9-8

63 Boscart VM, Fernie GR, Lee JH, *et al.* Using psychological theory to inform methods to optimize the implementation of a hand hygiene intervention. *Implement Sci* 2012;**7**:77. doi:10.1186/1748-5908-7-77

64 Finch T. Teledermatology for chronic disease management: coherence and normalization. *Chronic Illn* 2008;**4**:127–34. doi:10.1177/1742395308092483

65 May C. Towards a general theory of implementation. *Implement Sci* 2013;**8**:18. doi:10.1186/1748-5908-8-18

66 NPT Bibliography. http://www.normalizationprocess.org/bibliography/ (accessed 25 Feb2016).

67 BNF Publications. https://www.bnf.org/ (accessed 26 May2016).

68 O'Mahony D, O'Sullivan D, Byrne S, *et al.* STOPP/START criteria for potentially inappropriate prescribing in older people: version 2. *Age Ageing* 2015;**44**:213–8. doi:10.1093/ageing/afu145

69 Prescribing Decision Support for the NHS by ScriptSwitch | Better Prescribing | Clinical system prescribing software | Cost effective prescribing | Drug switches.

For peer review only

http://www.scriptswitch.com/ (accessed 22 May2017).

70 Guthrie B, Makubate B, Hernandez-Santiago V, *et al.* The rising tide of polypharmacy and drug-drug interactions: population database analysis 1995–2010. *BMC Med* 2015;**13**:74. doi:10.1186/s12916-015-0322-7

71 Moriarty F, Hardy C, Bennett K, *et al.* Trends and interaction of polypharmacy and potentially inappropriate prescribing in primary care over 15 years in Ireland: a repeated cross-sectional study. *BMJ Open* 2015;**5**:e008656. doi:10.1136/bmjopen-2015-008656

For peer review only

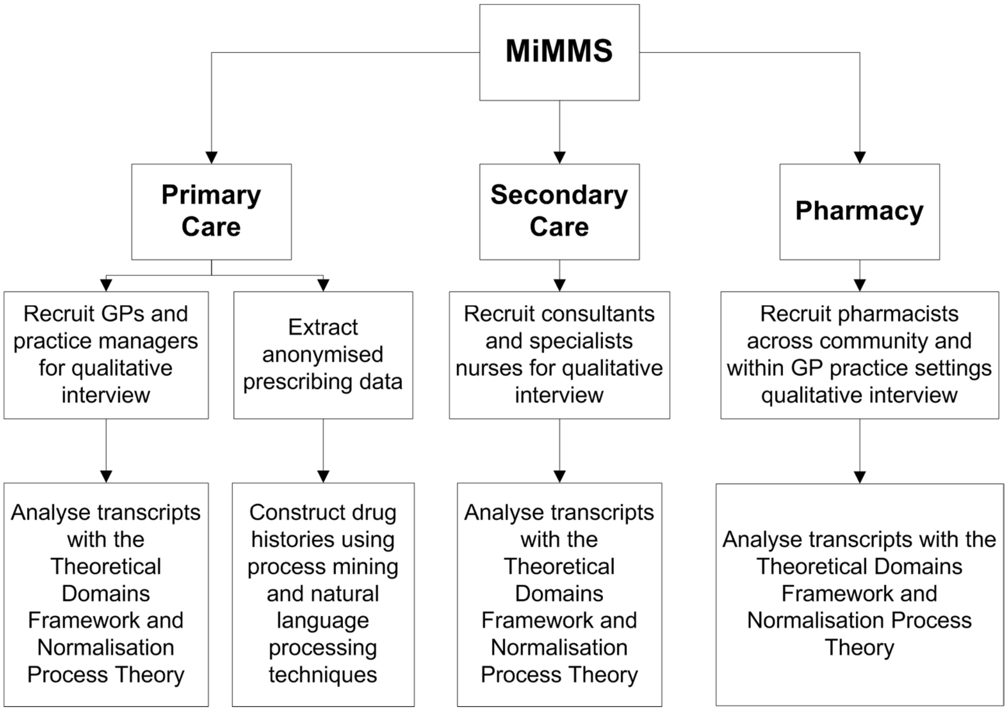

Figure 1: Flow diagram of MiMMS

156x110mm (300 x 300 DPI)

Supplementary file 1 Read codes

Condition	Included (including exception reporting)	Excluded
Hypertension	G2...<br>G20..%<br>G24.. - G2z.. (Excluding G24z1, G2400, G2410, G27..)<br>Gyu2.<br>Gyu20	21261<br>212K.
COPD	H3…<br>H31..% (excluding H3101, H31y0, H3122)<br>H32..%<br>H36.. - H3z.. (excluding H3y0., H3y1.)<br>H4640<br>H4641<br>H5832<br>Hyu30<br>Hyu31	2126F
Depression	E0013,<br>E0021<br>E112.%,<br>E113.%<br>E118.,<br>E11y2<br>E11z2,<br>E130.<br>E135.<br>E2003<br>E291.,<br>E2B..<br>E2B1.,<br>Eu204<br>Eu251<br>Eu32.% (excluding Eu32A, Eu32B, Eu329) Eu33.%<br>Eu341<br>Eu412	212S.
Diabetes	C10..,<br>C109J,<br>C109K,<br>C10C.,<br>C10D.,<br>C10E.%,<br>C10F.% (Excluding C10F8),	21263<br>212H.

For peer review only

	C10G.%,<br>C10H.%,<br>C10M.%,<br>C10N.%,<br>PKyP.,<br>C10P.%,<br>C10Q.	
Hypertension	G2...<br>G20..%<br>G24.. - G2z.. (Excluding G24z1, G2400, G2410, G27..)<br>Gyu2.<br>Gyu20	21261<br>212K.
Osteoarthritis	N05..00 Osteoarthritis and allied disorders	
CKD	1Z12.<br>1Z13.<br>1Z14.<br>1Z15.<br>1Z16.<br>1Z1B. - 1Z1L.<br>1Z1T.<br>1Z1V.<br>1Z1W.<br>1Z1X.<br>1Z1Y.<br>1Z1Z.<br>1Z1a.<br>1Z1b.<br>1Z1c.<br>1Z1d.<br>1Z1e.<br>1Z1f.<br>K053.<br>K054.<br>K055.<br>1Z10.<br>1Z11.<br>1Z17. - 1Z1A.<br>1Z1M.<br>1Z1Q.<br>K051.<br>K052.<br>1Z1N.<br>1Z1P.<br>1Z1R.<br>1Z1S.	2126E

For peer review only
